# EO-MTRNN: evolutionary optimization of hyperparameters for a neuro-inspired computational model of spatiotemporal learning

**DOI:** 10.1007/s00422-020-00828-8

**Published:** 2020-03-17

**Authors:** Erhard Wieser, Gordon Cheng

**Affiliations:** Institute for Cognitive Systems (ICS), Technical University of Munich, Munich, Germany

**Keywords:** EO-MTRNN, Autonomous hyperparameter estimation, Neural plasticity, Evolutionary optimization

## Abstract

For spatiotemporal learning with neural networks, hyperparameters are often set manually by a human expert. This is especially the case with multiple timescale networks that require a careful setting of the values of timescales in order to learn spatiotemporal data. However, this implies a cumbersome trial-and-error process until suitable parameters are found and it reduces the long-term autonomy of artificial agents, such as robots that are controlled by multiple timescale networks. To solve the problem, we propose the *evolutionary optimized multiple timescale recurrent neural network* (*EO-MTRNN*) that is inspired by the neural plasticity of the human cortex. Our proposed network uses a method of evolutionary optimization to adjust its timescales and to rewire itself in terms of number of neurons and synapses. Moreover, it does not require additional neural networks for pre- and postprocessing input–output data. We validate our EO-MTRNN by applying it to a proposed benchmark training dataset with single and multiple sequence training cases, as well as by applying it to sensory-motor data from a robot. We compare different configuration modes of the network, and we compare the learning performance between a network configuration with manually set hyperparameters and a configuration with automatically estimated hyperparameters. The results show that automatically estimated hyperparameters yield approximately 43% better performance than manually estimated ones, without overfitting the given teaching data. We also validate the generalization ability by successfully learning data that were not included in the hyperparameter estimation process.

## Introduction

It has become a matter of common knowledge that the human brain is highly adaptive. The neural structures in the neocortex can self-organize and rearrange themselves rapidly. This occurs especially in infancy, where there is a tremendous increase in the number of synapses and dendrites (Huttenlocher 1990). Cortical regions have the property of reshaping themselves in terms of not only increasing but also pruning the number of synaptic connections (Huttenlocher and Dabholkar 1997; Shaw et al. 2008). This restructuring happens over time over many years including adulthood. The more the structuring of a cortical region progresses over time, the more specific or specialized the region gets (Johnson 2010). The specialization that emerges over time seems not to be predetermined from the beginning on, but highly depends on the stimuli that it receives upon environmental interaction. This is referred to as *neuroplasticity* (Johnson and de Haan 2015). Modelling this plasticity in computational models such as artificial neural networks (ANNs) has not been well investigated in the past, since many hyperparameters representing structure and dynamics of the model are either predetermined, or manually changed during cumbersome trial-and-error experiments.

### Motivation

Our goal is to minimize the parameterization effort of a recurrent neural network (RNN) that is the multiple timescale recurrent neural network (MTRNN) originally proposed by Yamashita and Tani (2008). We take inspiration from the plasticity of the human cortex (Johnson and de Haan 2015; Johnson 2010). Further motivation comes from developmental robotics that is in turn influenced by neuroscientific insights (Lungarella et al. 2003).

The reconfiguration of system parameters by a human expert should be avoided during an agent’s run time. Agents that can autonomously adjust their parameters to learn new tasks are also characterized as *skull-closed* (Wang et al. 2011). This means that there is no need to stop the agent upon a problem, to open its settings, i.e. the “skull”, to make the changes or rewirings, and start it again in an effort that the behaviour will be better upon manual parameter change.

Likewise, it would be desirable if a neural network can restructure itself over time when the training data changes due to newly collected samples, e.g. when the network controls a robot.

### Problem

In detail, the original MTRNN (Yamashita and Tani 2008) is a special type of continuous time recurrent neural network (CTRNN) that operates with sigmoid activation in the fast and slow context group, and softmax activation in the input–output group. In the following paragraphs, we describe significant technical challenges of using the original MTRNN. Nevertheless, these challenges also apply to all future derivations of the MTRNN, such as an MTRNN with four different timescales (Alnajjar et al. 2013) and the MSTNN (Jung et al. 2015), i.e. to various recurrent neural networks with multiple scales.

**Finding the correct neural timescales** The learning performance of the MTRNN is strongly dependent on the settings of the timescales for each neuron group; in particular, the ratio between the timescales for fast context and slow context influences the performance (Yamashita and Tani 2008). Networks related to the MTRNN, such as the MSTNN (Jung et al. 2015), also rely on multiple timescales, and it is crucial to find good values for them. However, finding proper values for these different timescales is problematic, and so far it has been achieved through many trial-and-error tests conducted by a human experimenter who evaluated the network performance.

**Finding the number of context neurons** Determining the number of fast and slow context neurons is also not trivial. It is often dependent on the number of input–output neurons that in turn depend on the given pre- and postprocessing schema.

**Training additional neural networks for pre- and postprocessing** The MTRNN input and output are processed by topologically preserving maps (TPMs) (Kohonen 1982), like in Yamashita and Tani (2008) and Arie et al. (2012). This pre- and postprocessing of the network input and output increases the learning ability by reducing the overlaps in the training data (Yamashita and Tani 2008). However, the usage of TPMs represents a slight drawback of the original MTRNN, since the TPMs are neural networks that need to be trained as well in addition to the main network.

### Approach

In order to solve the problems, we propose to model the aforementioned neural plasticity by restructuring the network through changing the number of neurons of different groups and the corresponding number of synaptic weights. It is not enough to consider an optimization of the values of weights and initial neural potentials only, like it has been the case in many existing RNNs in the past. The number of neurons per neural group and the number of weights have to be optimized as well. In addition, the local activity of particular neurons has also to be optimized, a property that is crucial for learning to execute action sequences (Kiebel et al. 2008; Badre and D’Esposito 2009). We therefore optimize the timescale of each individual neuron. This timescale determines whether a neuron is *fast*, i.e. changing its activity quickly, or rather *slow*, i.e. changing its activity slowly.

In sum, we propose to optimize:

- Synaptic weights
- Initial potentials of context neurons
- Neural timescales
- Number of context neurons

Note that due to the connectivity of our network, an optimization of the number of context neurons implicitly also optimizes the number of synaptic weights.

**Fig. 1:**
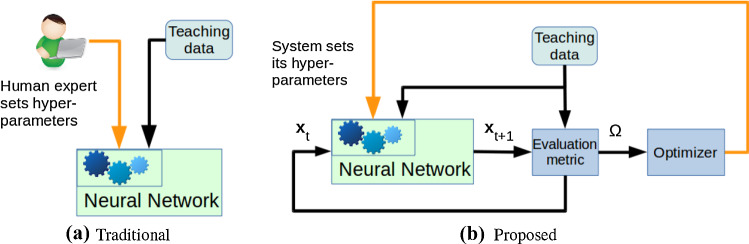
Traditional **a** a human determines the hyperparameters. This may imply a series of cumbersome trial-and-error runs, each run conducted with the human in the loop, until the system shows satisfactory learning performance. Proposed **b** System automatically determines its hyperparameters, i.e. neural dynamics and network structure, through a process resembling biological evolution, in which each generation of the network performs better than the previous one. For each generation, the performance is determined by running the network in closed-loop yielding spatiotemporal patterns $\mathbf {x}_0$, $\mathbf {x}_1$, ..., $\mathbf {x}_{L-1}$ that are compared with the teaching data. The resulting fitness metric $\varOmega$ is fed into the optimizer determining the hyperparameters for the next generation. To our best knowledge, evolutionary hyperparameter estimation has not yet been realized for *multiple timescale neural networks*

Formally, our proposed optimization schema can be expressed as

1 $$\begin{aligned} {\mathop {\hbox {argmax}}\limits _{{\varvec{\tau }}, \mathbf {n}}} \varOmega ({\varvec{\tau }}, \mathbf {n}, {\mathop {\hbox {argmin}}\limits _{\mathbf {W}, \mathbf {q}^\star }} E(\mathbf {W}, \mathbf {q}^\star )), \end{aligned}$$

where ${\varvec{\tau }}$, $\mathbf {n}$, and $\mathbf {q}^\star$ denotes the vector of all neural timescales, number of context neurons, and initial context potentials, respectively, and $\mathbf {W}$ denotes the matrix of synaptic weights; the function $\varOmega$ is a fitness function that is optimized via evolutionary optimization, while the error function *E* is optimized via backpropagation through time (BPTT) (Rumelhart et al. 1986, 2002). It is speculated that cortical regions also implement a form of BPTT (Whittington and Bogacz 2019). We adopt an evolutionary optimization method (Brest et al. 2006) realizing $\hbox {argmax}_{{\varvec{\tau }}, \mathbf {n}} \varOmega$ in order to model the plasticity of a cortical region, a process that happens over a longer timespan, e.g. over many developmental stages. One can relate a developmental stage to one or more *generations* of the optimization phase, where each generation has a particular fitness value $\varOmega$. In Fig. 1, we compare our proposed evolutionary approach with the traditional approach. We propose our version of the MTRNN that does *not* require additional training of subnetworks for pre- and postprocessing. This solves the latter challenge in the aforementioned problem section.

## Background

In Sect. 2.1, we provide an overview on recurrent neural networks that have multiple timescale properties emulating functional hierarchies in the neocortex. In order to overcome the aforementioned problems and to model the plasticity, we adopted a method of evolutionary optimization, a field that we briefly outline in Sect. 2.2.

### Recurrent neural networks with multiple timescales

In neuroscience, the theory of predictive coding (Friston 2008, 2010) attempts to model hierarchical message passing in the human cortex. Higher levels of the cortical hierarchy encode abstract entities such as goals or intentions (Fuster 2001; Badre and D’Esposito 2009). Representations in the higher levels are updated through the bottom-up error propagation process (Friston 2010). From a simplified point of view, each level of the cortical hierarchy operates with a particular timescale. This forms a functional hierarchy that can be best explained through the example of action selection and execution, referred to as *temporal abstraction* (Badre and D’Esposito 2009, p. 661).

In analogy to temporal abstraction, RNNs based on predictive coding have been studied by Tani (2016) in a robotics context. Compared to earlier RNNs, e.g. Elman (1990) and Jordan (1997), neural networks based on predictive coding have more than one timescale in their context group. Yamashita and Tani (2008) proposed the MTRNN that has two different timescales within its context group. The MTRNN is the foundation for newer networks that are based on the principle of multiple timescales, such as the MSTNN (Jung et al. 2015). The MTRNN overcomes the limited storage capacity of a traditional continuous time recurrent neural network (Tani et al. 2008). While the context group of traditional RNNs can encode goal-directed actions (Nishimoto et al. 2008), the switching or transition between different action primitives should happen smoothly and be also represented in the network. Instead of having many distinct local networks encoding primitive sequences and a higher-level selection module to switch between the output of these networks, the MTRNN integrates these capabilities in one single neural network through its multiple timescale property. Primitive sequences are encoded by a group of context neurons with a faster change in activity (fast context) compared to another group of context neurons with a slower change in activity (slow context). The slow context neurons alter their activity when a switching between primitive sequences occurs, which is, for example, the case at branching points of trajectories in space-time.

The application possibilities offered by the MTRNN as a dynamic system and the idea of multiple timescales have been further investigated in Arie et al. (2012), Yamashita and Tani (2012) and Jeong et al. (2012). Further MTRNN applications were shown in Sasaki et al. (2015), Takahashi et al. (2015b) and Takahashi et al. (2015a).

### Evolutionary optimization

We consider optimization methods that neither depend on an analytical description of the optimization problem, nor on its gradient. Evolutionary optimization has become increasingly popular, since it does not require information about the fitness landscape, works in high-dimensional search spaces, and can be parallelized (Yao and Xu 2006). Examples include random search (Anderson 1953; Solis and Wets 1981; Bergstra and Bengio 2012), although not necessarily considered as evolutionary. An optimization method for very large search spaces is particle swarm optimization (PSO) (Kennedy and Eberhart 1995; Eberhart and Kennedy 1995; Shi and Eberhart 1998). However, it is not guaranteed that PSO finds an optimal solution, and often, the user has to make a trade-off between exploration and exploitation (Trelea 2003). A promising candidate for global optimization is differential evolution (DE) (Storn and Price 1995; Storn 1996; Storn and Price 1997). Similar to PSO, DE can be applied to a wide variety of numerical optimization problems with very high dimensions, noise, and fluctuations over time, such as in Rocca et al. (2011). In Vesterstrom and Thomsen (2004) and Brest et al. (2006), it is reported that DE outperforms PSO in the quality of computed solutions on benchmark problems. However, as it is the case with other optimization methods, the performance of DE is dependent on its control parameters. Adverse values for these parameters deteriorate the optimization performance. To overcome this drawback, Liu and Lampinen (2005) proposed a fuzzy adaptive version of DE (FA-DE), which outperformed the original DE. Brest et al. (2006) proposed a version of DE with self-adapting control parameters (SA-DE), and they extensively compared the performance of SA-DE with FA-DE and other related methods. They reported that SA-DE yielded better results than FA-DE, and that SA-DE yielded better or at least comparable results than the other evolutionary algorithms proposed in Yao et al. (1999) and Lee and Yao (2004).

These findings suggest that SA-DE is the ideal candidate for the optimization of MTRNN hyperparameters.

## Computational model

### Acronyms and mathematical notations

We use the following acronyms unless defined otherwise:

IO: input–output
*C*: context, can be split into fast context (FC) and slow context (SC)
$$N_\mathrm{IO} \in \mathbb {N}$$:
number of input–output units
$$N_\mathrm{FC} \in \mathbb {N}$$:
number of fast context units
$$N_\mathrm{SC} \in \mathbb {N}$$:
number of slow context units
$$N_\mathrm{S} \in \mathbb {N}$$:
number of sequences
$$L \in \mathbb {N}$$:
sequence length
$$E \in \mathbb {R}$$:
loss function
$$\hat{y} \in \mathbb {R} | 0.0< \hat{y} < 1.0$$:
sample value (of a spatial dimension) of a training sequence
$$y \in \mathbb {R} | 0.0< y < 1.0$$:
activation value of an input–output unit
$$u \in \mathbb {R}$$:
potential value of an input–output unit
$$x \in \mathbb {R} | 0.0< x < 1.0$$:
input value fed into an input–output unit
$$c \in \mathbb {R} | 0.0< c < 1.0$$:
activation value of a context unit
$$q \in \mathbb {R}$$:
potential value of a context unit
$$\tau \in \mathbb {N}_{>0}$$:
timescale of a unit
$$w_{ab} \in \mathbb {R}$$:
connective weight from unit *b* to unit *a*
$$t \in \mathbb {N}$$:
timestep

### Network structure

**Fig. 2:**
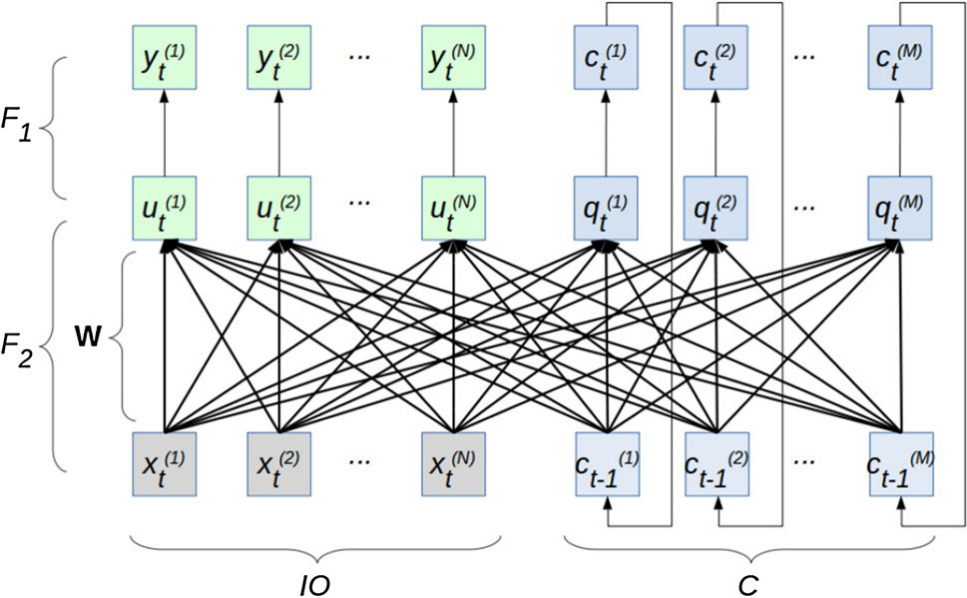
Structure of our MTRNN version that works with sigmoid activation for all units. The network consists of units (depicted as squares) connected by a set of mathematical functions $F_1$ and $F_2$. The left half shows the input–output group IO, and the right half shows the context group *C*. The thick arrows between the bottom and the middle units indicate the connective weights represented by the weight matrix $\mathbf {W}$. The output of the top context units is fed back to the bottom context units. The extension of this network by an evolutionary optimizer yields our proposed EO-MTRNN that is shown in Fig. 3

Figure 2 shows the structure of our proposed version of the MTRNN. The set $F_1$ represents the neural activation functions which are *y*(*u*) for the IO group and *c*(*q*) for the *C* group. The cardinality of $F_1$ is denoted as $|F_1|$. It is $|F_1| = N_\mathrm{IO} + N_\mathrm{FC} + N_\mathrm{SC}$. The set $F_2$ represents the functions for updating the neural potentials which are $u_t(\tau , u_{t-1}, w, x_t, c_{t-1})$ for the IO group and $q_t(\tau , q_{t-1}, w, x_t, c_{t-1})$ for the *C* group. The set $F_2$ has the same cardinality than $F_1$. The $F_2$ functions include recurrent dependencies, weights, and timescales. Note that the timescales can be different from each other, splitting the *C* group into FC and SC. Also note that the connections between the IO group and the SC group are set to zero.

One difference compared to the original version (Yamashita and Tani 2008) is the usage of sigmoid activation for the input–output group of the network, in order to keep it consistent with the activation for the context group having sigmoid activation as well. The entire network now consists of units with sigmoid activation. Besides uniformity, we decided to use the sigmoid activation for the input–output group because of the universal approximation theory of neural networks. Any continuous function can be approximated by sigmoid units (Cybenko 1989; Barron 1993). For a dynamic network, such as the MTRNN, this implies controllability, i.e. any desired state can be achieved within a finite number of steps starting from an initial state. The original version used softmax activation for the input–output group, since, as described in Yamashita and Tani (2008), the softmax activation fits well to the pre- and postprocessor networks that are TPMs (Kohonen 1982), connected to the MTRNN. However, these pre- and postprocessor networks have to be trained as well, in addition to the main network. In contrast, our version does not require pre- and postprocessor networks. Nevertheless, if pre- and postprocessing is requested to introduce a sparse representation of the actual input data, we can achieve it by a simple analytical pre- and postprocessing scheme. We propose this analytical preprocessing scheme as one of our network configuration modes. The second proposed mode is an early stopping method included into the training procedure. When activated, it is supposed to reduce the overfitting of the teaching data.

### Optimization of synaptic weights and initial neuron potentials

Here, we describe $\hbox {argmin}_{\mathbf {W}, \mathbf {q}^\star } E(\mathbf {W}, \mathbf {q}^\star )$ of Eq. (1).

We use sigmoid neurons throughout the entire network. The activation of an input–output neuron is given by Eq. (2) and the activation of a context neuron is given by Eq. (3):

2 $$\begin{aligned} y(u)= & {} \frac{1}{1+\exp (-u)} \end{aligned}$$

3 $$\begin{aligned} c(q)= & {} \frac{1}{1+\exp (-q)} . \end{aligned}$$

The potential value of an input–output unit is updated by

4 $$\begin{aligned}&u_t^{(i)} = \left( 1 - \frac{1}{\tau ^{(i)}}\right) u_{t-1}^{(i)} \nonumber \\&\quad \quad \,\,\, + \frac{1}{\tau ^{(i)}} \left( \sum _{k \in \mathrm{IO}} w_{ux}^{(ik)} x_t^{(k)} + \sum _{k \in C} w_{uc}^{(ik)} c_{t-1}^{(k)}\right) \end{aligned}$$

with $$i \in $$ IO.

The potential value of a context unit is updated by

5 $$\begin{aligned}&q_t^{(i)} = \Big (1 - \frac{1}{\tau ^{(i)}}\Big ) q_{t-1}^{(i)}\nonumber \\&\quad \quad \,\,\, + \frac{1}{\tau ^{(i)}} \left( \sum _{k \in \mathrm{IO}} w_{qx}^{(ik)} x_t^{(k)} + \sum _{k \in C} w_{qc}^{(ik)} c_{t-1}^{(k)}\right) \end{aligned}$$

with $$i \in C$$.

We use the following loss function:

6 $$\begin{aligned} E = \sum _t E_t = \sum _t \sum _{i \in IO} \frac{1}{2} \Big (\hat{y}_t^{(i)} - y_t^{(i)}\Big )^2 \end{aligned}$$

In the following, we derive all partial derivatives that are required for the update of synaptic weights and the update of initial potentials of context neurons. This derivation is based on the network structure (Fig. 2), the functions (2), (3), (4), (5), and the loss function (6). The partial derivative $\frac{\partial E}{\partial u_t^{(i)}}$ can be expressed as:

7 $$\begin{aligned} \frac{\partial E}{\partial u_t^{(i)}} = \frac{\partial E_t}{\partial u_t^{(i)}} + \frac{\partial }{\partial u_t^{(i)}} \left( \sum _{t'=t+1} E_{t'} \right) . \end{aligned}$$

Equation (7) is the recurrence equation of the input–output group, and it is developed to contain the recurrence term $\frac{\partial E}{\partial u_{t+1}^{(i)}} \frac{\partial u_{t+1}^{(i)}}{\partial u_t^{(i)}}$:

8 $$\begin{aligned} \frac{\partial E}{\partial u_t^{(i)}} = \frac{\partial E_t}{\partial u_t^{(i)}} + \frac{\partial E}{\partial u_{t+1}^{(i)}} \frac{\partial u_{t+1}^{(i)}}{\partial u_t^{(i)}} \end{aligned}$$

with $$i \in $$ IO.

The right side of Eq. (8) is going to be expanded. Applying the chain rule to $\frac{\partial E_t}{\partial u_t^{(i)}}$ and deriving $\frac{\partial u_{t+1}^{(i)}}{\partial u_t^{(i)}}$ by using Eq. (4) result in

9 $$\begin{aligned} \frac{\partial E}{\partial u_t^{(i)}} = \frac{\partial E_t}{\partial y_t^{(i)}} \frac{\partial y_t^{(i)}}{\partial u_t^{(i)}} + \frac{\partial E}{\partial u_{t+1}^{(i)}} \Big ( 1 - \frac{1}{\tau ^{(i)}} \Big ) \end{aligned}$$

It follows for one unit $i \in$ IO:

10 $$\begin{aligned} \frac{\partial E_t}{\partial y_t^{(i)}} = \frac{\partial }{\partial y_t^{(i)}} \bigg ( \frac{1}{2} \Big ( \hat{y}_t^{(i)} - y_t^{(i)} \Big )^2 \bigg ) = - \Big ( \hat{y}_t^{(i)} - y_t^{(i)} \Big ) \end{aligned}$$

The variable $y_t^{(i)}$ denotes the sigmoid activation of the IO group (Eq. (2)); thus,

11 $$\begin{aligned} \frac{\partial y_t^{(i)}}{\partial u_t^{(i)}} = \frac{\partial }{\partial u_t^{(i)}} \Bigg ( \frac{1}{1+\exp (-u_t^{(i)})} \Bigg ) = y_t^{(i)} \Big ( 1 - y_t^{(i)} \Big ).\nonumber \\ \end{aligned}$$

Inserting Eq. (10) and Eq. (11) back into Eq. (9) yields

12 $$\begin{aligned} \frac{\partial E}{\partial u_t^{(i)}} = \Big ( y_t^{(i)} - \hat{y}_t^{(i)} \Big ) y_t^{(i)} \Big ( 1 - y_t^{(i)} \Big ) + \Big ( 1 - \frac{1}{\tau ^{(i)}} \Big ) \frac{\partial E}{\partial u_{t+1}^{(i)}} \end{aligned}$$

with $$i \in $$ IO.

In analogy to Eq. (7), the partial derivative $\frac{\partial E}{\partial q_t^{(i)}}$ can be expressed as

13 $$\begin{aligned} \frac{\partial E}{\partial q_t^{(i)}} = \sum _{k \in \mathrm{IO}} \frac{\partial E}{\partial u_{t+1}^{(k)}} \frac{\partial u_{t+1}^{(k)}}{\partial c_t^{(i)}} \frac{\partial c_t^{(i)}}{\partial q_t^{(i)}} + \sum _{k \in C} \frac{\partial E}{\partial q_{t+1}^{(k)}} \frac{\partial q_{t+1}^{(k)}}{\partial q_t^{(i)}} \end{aligned}$$

with $i \in C$. Equation (13) is the recurrence equation of the context group, resulting from the structure and connectivity of the network. The right side of Eq. (13) contains the following parts:

14 $$\begin{aligned} \frac{\partial u_{t+1}^{(k)}}{\partial c_t^{(i)}} = \frac{1}{\tau ^{(k)}} w_{uc}^{(ki)} \end{aligned}$$

with $i \in C$ and $k \in$ IO,

15 $$\begin{aligned} \frac{\partial c_t^{(i)}}{\partial q_t^{(i)}} = \frac{\partial }{\partial q_t^{(i)}} \Bigg ( \frac{1}{1+\exp (-q_t^{(i)})} \Bigg ) = c_t^{(i)} \Big ( 1 - c_t^{(i)} \Big ) \end{aligned}$$

with $$i \in C$$,

16 $$\begin{aligned} \frac{\partial q_{t+1}^{(k)}}{\partial q_t^{(i)}} = \delta _{ik} \Big ( 1 - \frac{1}{\tau ^{(k)}} \Big ) + \frac{1}{\tau ^{(k)}} w_{qc}^{(ki)} \frac{\partial c_t^{(i)}}{\partial q_t^{(i)}} \end{aligned}$$

with $i \in C$, $k \in C$, and $\delta _{ik}$ as Kronecker delta ($\delta _{ik} = 1$ for $i = k$, $\delta _{ik} = 0$ for $i \ne k$). Inserting Eq. (15) into Eq. (16) results in

17 $$\begin{aligned} \frac{\partial q_{t+1}^{(k)}}{\partial q_t^{(i)}} = \delta _{ik} \Big ( 1 - \frac{1}{\tau ^{(k)}} \Big ) + \frac{1}{\tau ^{(k)}} w_{qc}^{(ki)} c_t^{(i)} \Big ( 1 - c_t^{(i)} \Big ) \end{aligned}$$

with $i \in C$ and $k \in C$. Inserting Eq. (14), Eqs. (15), and (17) back into Eq. (13) yields

18 $$\begin{aligned} \frac{\partial E}{\partial q_t^{(i)}}&= \sum _{k \in \mathrm{IO}} \frac{\partial E}{\partial u_{t+1}^{(k)}} \frac{1}{\tau ^{(k)}} w_{uc}^{(ki)} c_t^{(i)} \Big (1 - c_t^{(i)}\Big ) \nonumber \\&\quad + \sum _{k \in C} \frac{\partial E}{\partial q_{t+1}^{(k)}} \bigg ( \delta _{ik}\Big (1 - \frac{1}{\tau ^{(k)}}\Big ) \nonumber \\&\quad + \frac{1}{\tau ^{(k)}} w_{qc}^{(ki)} c_t^{(i)} \Big (1 - c_t^{(i)}\Big ) \bigg ) \end{aligned}$$

with $i \in C$. The partial derivatives $\frac{\partial E}{\partial u_t^{(i)}}$ (Eq. (12)) and $\frac{\partial E}{\partial q_t^{(i)}}$ (Eq. (18)) are important, since they are required to compute the gradients $\frac{\partial E}{\partial w}$ according to:

19 $$\begin{aligned} \frac{\partial E}{\partial w_{ux}^{(ik)}} = \sum _t \frac{\partial E}{\partial u_t^{(i)}} \frac{\partial u_t^{(i)}}{\partial w_{ux}^{(ik)}} = \sum _t \frac{\partial E}{\partial u_t^{(i)}} \frac{1}{\tau ^{(i)}} x_t^{(k)} \end{aligned}$$

with $$i, k \in $$ IO,

20 $$\begin{aligned} \frac{\partial E}{\partial w_{uc}^{(ik)}} = \sum _t \frac{\partial E}{\partial u_t^{(i)}} \frac{\partial u_t^{(i)}}{\partial w_{uc}^{(ik)}} = \sum _t \frac{\partial E}{\partial u_t^{(i)}} \frac{1}{\tau ^{(i)}} c_{t-1}^{(k)} \end{aligned}$$

with $$i \in \hbox {IO}, k \in C$$,

21 $$\begin{aligned} \frac{\partial E}{\partial w_{qx}^{(ik)}} = \sum _t \frac{\partial E}{\partial q_t^{(i)}} \frac{\partial q_t^{(i)}}{\partial w_{qx}^{(ik)}} = \sum _t \frac{\partial E}{\partial q_t^{(i)}} \frac{1}{\tau ^{(i)}} x_t^{(k)} \end{aligned}$$

with $$i \in C, k \in $$ IO,

22 $$\begin{aligned} \frac{\partial E}{\partial w_{qc}^{(ik)}} = \sum _t \frac{\partial E}{\partial q_t^{(i)}} \frac{\partial q_t^{(i)}}{\partial w_{qc}^{(ik)}} = \sum _t \frac{\partial E}{\partial q_t^{(i)}} \frac{1}{\tau ^{(i)}} c_{t-1}^{(k)} \end{aligned}$$

with $$i \in C, k \in C$$.

Given these partial derivatives, the weights and initial potentials can be updated as part of the BPTT algorithm (Rumelhart et al. 1986, 2002). Within BPTT, the partial derivatives (12), (18) are iteratively computed for each training sequence and are required to compute the partial derivatives of the weights. The partial derivatives (19), (20) (21), (22) are iteratively computed for each training sequence with *t* as sample index, and then, they are summed up over the training sequences *s* given in the training set *S*.

At the beginning of training, the procedure initializes the weights with random values between $-\,0.025$ and 0.025 like in Yamashita and Tani (2008). In the multiple timescale mode, i.e. $\tau _\mathrm{FC} \ne \tau _\mathrm{SC}$, the connective weights between the IO group and the SC group are set to zero. The weights are updated by:

23 $$\begin{aligned} \varDelta w_{n+1}&= \alpha \frac{1}{T} \sum _{s \in S} \frac{\partial E^{(s)}}{\partial w} + \eta \varDelta w_n \nonumber \\ w_{n+1}&= w_n - \varDelta w_{n+1} \end{aligned}$$

where *n* is the epoch index, *T* is the number of total samples of the training set, $\alpha$ is the learning rate of weight update, and $\eta$ is the momentum. When the BPTT procedure arrives at the very first sample of a sequence, an additional backpropagation step is required in order to compute the initial potentials of the context neurons:

24 $$\begin{aligned} \frac{\partial E}{\partial q^{\star (i)}}&= \sum _{k \in \mathrm{IO}} \frac{\partial E}{\partial u_{0}^{(k)}} \frac{1}{\tau ^{(k)}} w_{uc}^{(ki)} c^{\star (i)} \Big (1 - c^{\star (i)}\Big ) \nonumber \\&\quad + \sum _{k \in C} \frac{\partial E}{\partial q_{0}^{(k)}} \bigg ( \delta _{ik}\Big (1 - \frac{1}{\tau ^{(k)}}\Big ) \nonumber \\&\quad + \frac{1}{\tau ^{(k)}} w_{qc}^{(ki)} c^{\star (i)} \Big (1 - c^{\star (i)}\Big ) \bigg ) \end{aligned}$$

with $i \in C$ and $\delta _{ik}$ as Kronecker delta. In Eq. (24), $c^{\star (i)}$ represents the initial input activation value of a context neuron *i*. The value of $c^{\star (i)}$ is set to 0.5 before the training starts. An activation value of 0.5 corresponds to a potential of 0 for a sigmoid neuron. It is considered as a neutral value from which the initial potential is adapted through the training process. For the set *S* of given training sequences, the initial context potentials $q^\star$ are updated by

25 $$\begin{aligned} q_{n+1}^{\star (si)} = q_n^{\star (si)} - \beta ^{(i)} \frac{1}{L_\mathrm{s}} \frac{\partial E^{(s)}}{\partial q^{\star (i)}} \end{aligned}$$

where $s \in S$, $i \in C$, *n* is the epoch index, $L_\mathrm{s}$ is the sequence length, and $\beta ^{(i)}$ is the learning rate of potential update. Note that $\beta ^{(i)}$ can have two different values, depending on whether the unit *i* is part of the fast context or slow context group.

The training procedure also contains a slight bias by clipping the gradients (12), (18), (24) through $\delta _c := tanh(\delta )$ where in this case $\delta$ represents the gradient (12), (18), (24), respectively. The weight gradients (19), (20), (21), (22) as well as the initial potentials (25) are then computed using the corresponding clipped gradient $\delta _c$. The usage of clipped gradients stabilizes the training procedure by preventing an accumulation of too high values for the partial derivatives (Goodfellow et al. 2016).

We used the mean squared error (MSE) as part of the BPTT training procedure. The reason for choosing the MSE resides in machine learning theory. The basis for supervised learning is the maximum likelihood estimator that aims to maximize a log-likelihood. Maximizing the log-likelihood with respect to the model parameters yields the same estimate of parameters than minimizing the MSE (Goodfellow et al. 2016, p.132).

In the recall phase after training, when an input sample is fed into the input–output group of the network, the entire context states, i.e. fast and slow context, are recognized through an iterative value search and initialized. Given a particular sequence $S_g$ and an input pattern $\mathbf {x}_t$, the context recognition starts with setting the initial IO and the initial *C* activation states from the BPTT training procedure. By closed-loop prediction, where the network output is fed into the input, subsequent IO patterns are computed, each with its corresponding *C* activation. The predicted IO pattern that has the minimum Euclidian distance to the given input pattern $\mathbf {x}_t$ is selected, and its corresponding *C* activation is retrieved. With the retrieved context activation, the sequence can then be predicted, i.e. generating $\mathbf {x}_{t+1}, \mathbf {x}_{t+2}$, etc., by using the learned weights and the neural updates according to Eqs. (2), (3), (4), (5).

### Early stopping

An optional part of the learning is an early stopping method. We divided the entire set of teaching data into training set and validation set. The training set is fed into the BPTT used to alter the weights and initial context potentials, and the validation set is used for early stopping. Given a set of sequences as teaching data, every third sample of a sequence was excluded from training and used for validation, yielding a data division of $67\%$ for training and $33\%$ for validation. For each epoch of training, the early stopping method does forward propagation and computes the MSE on the validation set. The method keeps a history of the validation MSE together with the weights and initial context potentials for the latest $\varDelta h$ epochs where $\varDelta h$ is the size of the epoch window. During the training process, if the MSE on the training set becomes smaller than a defined minimum value (in our case 0.0009), the method computes the gradient $g_\mathrm{v}$ of the validation MSE according to

26 $$\begin{aligned} g_\mathrm{v} = \frac{\varDelta e_\mathrm{v}}{\varDelta h} = \frac{e_\mathrm{v}(h) - e_\mathrm{v}(h-\varDelta h)}{\varDelta h} \end{aligned}$$

where $e_\mathrm{v}(h)$ is the validation MSE at the current epoch *h*. We set $\varDelta h$ to 500. During the training process, the validation MSE declines together with the training MSE. However, if the validation MSE starts to rise ($g_\mathrm{v} > 0$, which means $\varDelta e_\mathrm{v} > 0$), then the training is stopped and the weights and initial context potentials, which correspond to the minimum validation MSE, are returned.

### Input–output preprocessing

In the default configuration, each dimension of an input sample is mapped to one IO neuron. For example, if the network is supposed to learn eight-dimensional sequences, then the number of IO neurons will be eight. An alternative configuration is an input–output preprocessing, where each dimension of an input sample at timestep *t* is mapped to more than one IO neuron. Likewise, after the network computed the prediction at $t+1$, the activation of a number of IO neurons is mapped back to one dimension of the output sample. For a given input sample $\mathbf {x}$ with the dimension *m*, input mapping is done by $\mathbf {X} = \mathbf {x} \cdot \mathbf {v}^T$, where $\mathbf {v}$ is the preprocessing weight vector with the dimension *n*. The preprocessing vector has the elements $v_k \in \mathbb {R}$ | $0.0< v_k < 1.0$. When applying the preprocessing, the number of IO neurons required is $m \cdot n$. Backward mapping is done by $(\sum _k X_{ik}/v_k)/\hbox {dim}(\mathbf {v})$ for each *i*, where $X_{ik}$ is an element of $\mathbf {X}$, $v_k$ is an element of $\mathbf {v}$, with *i* as dimension index of the input pattern, and *k* is the dimension index of the preprocessing weight vector. Unless defined otherwise, we used $$\mathbf {v}^T = \begin{bmatrix} 0.225&0.45&0.9&0.45&0.225 \end{bmatrix}$$, yielding a pyramid-like activation pattern over each cluster of *n* IO neurons. The values of $\mathbf {v}$ were determined empirically, and they can be different. The middle element should have a value close to 1.0, and the values of the adjacent elements should be significantly smaller. This yields a sparse activation of the IO neurons of the network.

**Fig. 3:**
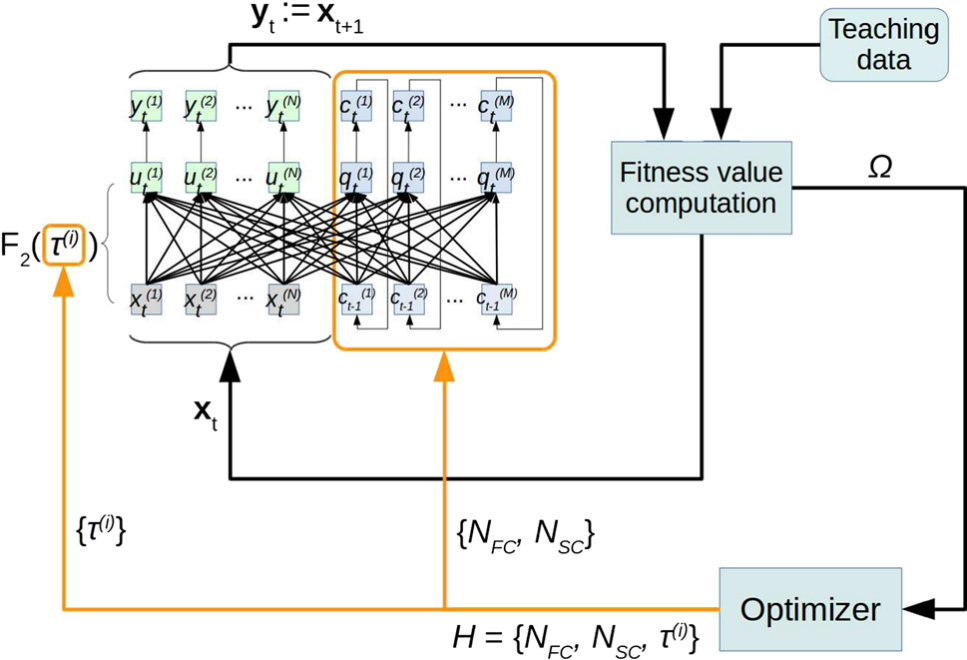
Proposed EO-MTRNN. The MTRNN (top left) is extended by components for autonomous hyperparameter estimation. For this purpose, the MTRNN is trained and evaluated with benchmark sequences (*teaching data*). The fitness $\varOmega$ is computed and fed into the *optimizer* that estimates a set *H* of hyperparameters. Here, *H* consists of the number of context neurons $N_\mathrm{FC}$, $N_\mathrm{SC}$, and the multiple timescales $\tau ^{(i)}$ with $i \in$ IO, FC, SC. The hyperparameters are used to adjust the neural timescales and to restructure the MTRNN (timescale adjustment and restructuring indicated by the orange colour) (colour figure online)

### Structure of the network extended by an evolutionary optimizer

Figure 3 shows the structure of our proposed EO-MTRNN: the MTRNN extended by components for autonomous hyperparameter estimation (AHE). Besides a given set of teaching data, the structure consists of three main components:

- The MTRNN (Sect. 3.2)
- Evaluation metric that is the computation of a fitness value
- Optimizer, i.e. an evolutionary algorithm that is the SA-DE

The aim is to improve the learner performance by maximizing a fitness value $\varOmega$. A generation of the network is represented by *H*, its weights $\mathbf {W}$, and initial context potentials $\mathbf {q}^*$. For each generation, the *optimizer* trains the MTRNN with the current population as well as with a mutated population of hyperparameters. For each individual of the population, a mutated individual of *H* will replace a population individual, if the mutated individual has a higher fitness value. Through this process, the *optimizer* creates a new generation of the network that has a higher fitness, i.e. better performance, than the previous one.

### Fitness value computation

The goal of the fitness value computation is to establish a mapping $$\varOmega $$ from a vector $$\mathbf {p}$$$$\in {\mathbb {N}}^{D_\mathrm{p}}$$ to a real scalar value, mathematically $$\varOmega : \mathbf {p} \rightarrow \mathbb R$$. The vector $$\mathbf {p}$$ has the dimension $$D_\mathrm{p}$$ (problem dimension) and represents the current setting of hyperparameters that are used for the optimization process. In case of the MTRNN hyperparameters, the vector $$\mathbf {p}$$ contains integer values. Table 1 shows the MTRNN hyperparameters which are optimized dependent on the value of the problem dimension $$D_\mathrm{p}$$. The default setting is $$D_\mathrm{P} = 5$$, and then, the number of all context neurons and all timescales are optimized. The other settings ($$D_\mathrm{P} < 5$$) are optional, and they can be used to customize the network optimization, for example if the user only wants to optimize one or more particular hyperparameter(s), while the others stay fixed.

**Table 1:** Dimension of optimization problem and the corresponding MTRNN hyperparameters that are optimized through SA-DE

Problem dimension $D_\mathrm{P}$	Hyperparameter *H*
1	$\tau _\mathrm{SC}$
2	$\tau _\mathrm{FC}$, $\tau _\mathrm{SC}$
3	$\tau _\mathrm{IO}$, $\tau _\mathrm{FC}$, $\tau _\mathrm{SC}$
4	$N_\mathrm{SC}$, $\tau _\mathrm{IO}$, $\tau _\mathrm{FC}$, $\tau _\mathrm{SC}$
5	$N_\mathrm{FC}$, $N_\mathrm{SC}$, $\tau _\mathrm{IO}$, $\tau _\mathrm{FC}$, $\tau _\mathrm{SC}$

For any particular setting of hyperparameters, the MTRNN is trained with a set of sequences (represented by the box *teaching data* in Fig. 3). It does not matter where the teaching data come from, at least for the scope of this paper. It can be either the set of benchmark sequences, or any other data set containing spatiotemporal patterns, e.g. sensory-motor data collected on a robot. The mapping $\varOmega$ represents an evaluation metric and is considered as fitness value for the hyperparameter optimization process. This fitness value is computed by using the training data and the current network output. We use the normalized sum of the entries of the *S* x *B* matrix of correlation coefficients:

27 $$\begin{aligned} \varOmega := \frac{1}{S \cdot B} \sum _{i=1}^S \sum _{j=1}^B r_{ij}, \end{aligned}$$

where *S* is the number of training sequences and *B* is the number of spatial dimensions of the sequences. Each entry $$r_{ij}$$$$\in \mathbb {R}$$ | $$-1.0 \le r_{ij} \le 1.0$$ is computed by:

28 $$\begin{aligned} r_{ij} = \frac{\sum _{t=1}^L (a_t^{(ij)} - \bar{a}^{(ij)}) (y_t^{(ij)} - \bar{y}^{(ij)})}{\sqrt{\sum _{t=1}^L (a_t^{(ij)} - \bar{a}^{(ij)})^2} \sqrt{\sum _{t=1}^L (y_t^{(ij)} - \bar{y}^{(ij)})^2}}, \end{aligned}$$

where $$a_t^{(ij)}$$$$\in \mathbb {R}$$ | $$0.0< a_t^{(ij)} < 1.0$$ denotes the value of a training sample at timestep *t* of sequence *i* and its spatial dimension *j*. Accordingly, $$y_t^{(ij)}$$$$\in \mathbb {R}$$ | $$0.0< y_t^{(ij)} < 1.0$$ denotes the value of a predicted sample at timestep *t* of sequence *i* and its spatial dimension *j*. The mean value of sequence *i* and its spatial dimension *j* are given by $$\bar{a}^{(ij)}$$ and $$\bar{y}^{(ij)}$$ for training and prediction, respectively. The values $$y_t^{(ij)}$$ are obtained by running the MTRNN in closed loop as indicated in Fig. 3.

### Optimization of timescales and number of neurons

Given the result of ${\hbox {argmin}_{\mathbf {W}, \mathbf {q}^\star } E}$, we describe $\hbox {argmax}_{{\varvec{\tau }}, \mathbf {n}} \varOmega$ of Eq. (1).

Using $\varOmega$ as optimization metric, the optimizer computes a new set of hyperparameters *H* which is used to restructure or rewire the spatiotemporal learner, i.e. the MTRNN. In this case, restructuring means altering the timescales which effect the dynamics and altering the number of context neurons, both fast and slow context. To this end, we adopted the method of differential evolution with autonomous meta-parameter^1^ adaptation proposed in Brest et al. (2006). It extends the original differential evolution (Storn and Price 1997) by making its meta-parameters self-adapting. In order to find suitable MTRNN hyperparameters, we applied SA-DE because it offers a global optimization as well as autonomous meta-parameter adaptation. Thus, the SA-DE is an enhanced version of the DE that already finds a *global* optimum over continuous spaces (Brest et al. 2006, p. 647). Using an optimizer with autonomous meta-parameter adaptation is beneficial, since the optimal meta-parameter settings are problem dependent (Brest et al. 2006).

The goal of this optimization is to maximize $\varOmega$. The optimization process works with individuals of two types of populations: the original population and the crossover population. Following the notation in Sect. 3.7, an individual *i* of the original population is described by the vector $\mathbf {p}_i$ containing hyperparameters. Additionally, an individual *i* of the crossover population is described by the vector $\mathbf {c}_i$. Both populations have the same size NP, thus *i* going from 1 to NP. For example, if $\hbox {NP} = 4$, then the population consists of $\{ \mathbf {p}_1, \mathbf {p}_2, \mathbf {p}_3, \mathbf {p}_4 \}$. A property of the SA-DE method is that $\hbox {NP} \ge 4$. The population size is initialized only once and kept constant during the optimization process; we used $\hbox {NP} = 4$. The SA-DE method contains two meta-parameters that are self-adapting: the mutation control *F*$\in \mathbb {R}$, also called differential weight, and the crossover control CR $\in \mathbb {R}$. The adaptation of *F* is influenced by the lower bound $F_\mathrm{l}$ and the upper bound $F_\mathrm{u}$; the adaptation of CR is influenced by the probabilities $\tau _1$ and $\tau _2$. The parameters $F_\mathrm{l}$, $F_\mathrm{u}$, $\tau _1$, and $\tau _2$ are initialized only once and kept constant during the optimization process. We used the same values as in Brest et al. (2006): $F_\mathrm{l} = 0.1$, $F_\mathrm{u} = 0.9$, $\tau _1 = 0.1$, and $\tau _2 = 0.1$.

**Table 2:** Elementary benchmark training sequences with Gaussian noise $\eta$ with $\mu = 0$ and $\sigma = 0.01$

Type	Length *L*	Mathematical expression *y*(*t*)	
Rising ramp	50	$y_1 = 0.0196 t + 0.02 + \eta$	
	100	$y_2 = 0.0097 t + 0.02 + \eta$	
	150	$y_3 = 0.0064 t + 0.02 + \eta$	
Falling ramp	50	$y_4 = -\,0.0196 t + 0.98 + \eta$	
	100	$y_5 = -\,0.0097 t + 0.98 + \eta$	
	150	$y_6 = -\,0.0064 t + 0.98 + \eta$	
Sigmoid-like upw. slope	50	$y_7 = 0.9 \exp (-\,10 \exp (-\,0.2 t)) + 0.04 + \eta$	
	100	$y_8 = 0.9 \exp (-\,50 \exp (-\,0.1 t)) + 0.04 + \eta$	
	150	$y_9 = 0.9 \exp (-\,100 \exp (-\,0.06 t)) + 0.04 + \eta$	
Sigmoid-like downw. slope	50	$y_{10} = -\,0.9 \exp (-\,10 \exp (-\,0.2 t)) + 0.94 + \eta$	
	100	$y_{11} = -\,0.9 \exp (-\,50 \exp (-\,0.1 t)) + 0.96 + \eta$	
	150	$y_{12} = -\,0.9 \exp (-\,100 \exp (-\,0.06 t)) + 0.96 + \eta$	
Sine	50	$y_{13} = 0.35\sin (2\pi t) + 0.5 + \eta$	
	100	$y_{14} = 0.35\sin (2\pi t) + 0.5 + \eta$	
	150	$y_{15} = 0.35\sin (2\pi t) + 0.5 + \eta$	
Irregular (type K)	50	$0 \le t \le 20$:	$y_{16} = 0.5\exp (-\,5\exp (-\,0.6 t)) + 0.04 + \eta$
		$21 \le t \le 40$:	$y_{16} = 0.3\exp (-\,5\exp (-\,0.6 (t-21)) + 0.5 + \eta$
		$41 \le t < 50$:	$y_{16} = -\,0.3\exp (-\,5\exp (-\,0.6 (t-41))) + 0.5 + \eta$
	100	$0 \le t \le 50$:	Same as irregular (type K) with $L = 50$
		$51 \le t \le 70$:	$y_{17} = 0.3\exp (-\,10\exp (-\,0.6 (t-51))) + 0.2 + \eta$
		$71 \le t < 100$:	$y_{17} = -\,0.3\exp (-\,19\exp (-\,0.6 (t-71))) + 0.5 + \eta$
	150	$0 \le t \le 100$:	Same as irregular (type K) with $L = 100$
		$101 \le t \le 120$:	$y_{18} = 0.3\exp (-\,12\exp (-\,0.6 (t-101))) + 0.2 + \eta$
		$121 \le t < 150$:	$y_{18} = -\,0.3\exp (-\,12\exp (-\,0.4 (t-121))) + 0.5 + \eta$

The optimization process includes the following key steps:

1. *Initialize population*
  The first step of the algorithm is to initialize the original population with random positions in the search space. The search space is bounded by the vectors lower bound ${\mathbf {b}}_\mathrm{lo}$ and upper bound ${\mathbf {b}}_\mathrm{up}$, both of the problem dimension $D_\mathrm{P}$. The control parameters *F* and CR are extended to become vectors $\mathbf {f}$ and $\mathbf {cr}$, respectively, both with dimension $\hbox {NP}$. Each element of $\mathbf {f}$ is initialized with a random floating-point value between $F_\mathrm{l}$ and $F_\mathrm{u}$, i.e. $f_i \in [ F_\mathrm{l}, F_\mathrm{u} ]$, from uniform distribution. Each element of $\mathbf {cr}$ is initialized with a random floating-point value between 0 and 1, i.e. $\hbox {cr}_i \in [ 0.0, 1.0 ]$, from uniform distribution.
2. *Compute crossover individuals*
  For each individual, four random values $$r_1$$, $$r_2$$, $$r_3$$, $$r_4$$$$\in \mathbb {R}$$ are generated with $$r \in [ 0.0, 1.0 ]$$, from uniform distribution. Then, each element of the control parameter vectors is adapted according to the following rule:
  29 $$\begin{aligned} f_i^{(g+1)}= & {} {\left\{ \begin{array}{ll} F_\mathrm{l} + r_1 \cdot F_\mathrm{u}, &{} \text {if } r_2 < \tau _1.\\ f_i^{(g)}, &{} \text {otherwise}. \end{array}\right. } \end{aligned}$$
  30 $$\begin{aligned} \hbox {cr}_i^{(g+1)}= & {} {\left\{ \begin{array}{ll} r_3, &{} \text {if } r_4 < \tau _2.\\ \hbox {cr}_i^{(g)}, &{} \text {otherwise}. \end{array}\right. } \end{aligned}$$
  where *g*, $F_\mathrm{l}$, $F_\mathrm{u}$, $f_i$, and $\hbox {cr}_i$ denote the current generation, lower bound, upper bound, mutation control, and crossover control, respectively. The parameters $\tau _1$ and $\tau _2$ are probabilities for modifying $f_i$, and $\hbox {cr}_i$, respectively. This adaptation of control parameters according to Eqs. (29) and (30) is done *before* the next two steps, the mutation and the crossover.
  The mutation step and the crossover step are done for each individual *i* of the current population:
  The mutation step begins with picking three individuals *a*, *b*, *c* from the population at random; these three have to be different from each other and different from the current target individual *i* that is being updated, mathematically $i \ne a \ne b \ne c$. A mutated individual $\mathbf {m}_i$, with dimension $D_\mathrm{P}$, is computed by
  31 $$\begin{aligned} \mathbf {m}_i = \mathbf {p}_a + f_i \cdot (\mathbf {p}_b - \mathbf {p}_c) \end{aligned}$$
  with population individuals $\mathbf {p}_a$, $\mathbf {p}_b$, $\mathbf {p}_c$, and crossover control $f_i$. The mutation $\mathbf {m}_i$ is then pruned to be within the search space bounded by ${\mathbf {b}}_\mathrm{lo}$ and ${\mathbf {b}}_\mathrm{up}$.
  The crossover step begins with taking a random index $d_x$ in the problem dimension, which means $d_x$ is an integer with $d_x \in [ 1, ..., D_\mathrm{P} ]$. Then, the crossover individual is computed, which is a mixture of an original individual with the mutated individual. For each dimension $k \in [ 1, ..., D_\mathrm{P} ]$, a uniform random (floating-point) number $r_k$ is generated with $r_k \in [ 0.0, 1.0 ]$ and the vector element $c_{ik}$ of the crossover individual $\mathbf {c}_i$ is computed according to
  32 $$\begin{aligned} c_{ik} = {\left\{ \begin{array}{ll} m_{ik}, &{} \text {if } r_k \le \hbox {cr}_i \text { or } k = d_x \\ p_{ik}, &{} \text {if } r_k > \hbox {cr}_i \text { and } k \ne d_x \end{array}\right. } \end{aligned}$$
  with $m_{ik}$ as *k*-th element of the mutated individual $\mathbf {m}_i$ and $p_{ik}$ as *k*-th element of the population individual $\mathbf {p}_i$.
3. *Compute next generation*
  Based on the result of the previous computation of crossover individuals, this step implements a selection process that leads to a new population generation. In other words, it is the creation of a population $g+1$ based on the previous population *g*.
  We introduce $$v_i^{(\mathrm{P})}$$$$\in \mathbb {R}$$ and $$v_i^{\mathrm{(C)}}$$$$\in \mathbb {R}$$ as elements of the population fitness vector $${\mathbf {v}}^{(\mathrm{P})}$$ and crossover fitness vector $${\mathbf {v}}^{\mathrm{(C)}}$$, respectively. Both vectors have the dimension $$\hbox {NP}$$. The computation of any fitness value *v* is described in Sect. 3.7. Computation of $$v_i^{(\mathrm{P})}$$ requires a training of the MTRNN with the hyperparameters contained in the population individual $$\mathbf {p}_i$$. Computation of $$v_i^{\mathrm{(C)}}$$ requires a training of the MTRNN with the hyperparameters contained in the crossover individual $$\mathbf {c}_i$$.
  Differential evolution originally minimizes a fitness $\varOmega$. Maximization is done by setting $\varOmega ^* := -\varOmega$ and using the fitness $\varOmega ^*$ when computing the selection.
  In our case, this is implemented by the boolean variable *max* that was always *true*, since the goal is fitness maximization. For each individual *i*, the update is
  33 $$\begin{aligned} \left. \begin{aligned} v_i^{(\mathrm{P})} \leftarrow -v_i^{(\mathrm{P})} \\ v_i^{\mathrm{(C)}} \leftarrow -v_i^{\mathrm{(C)}} \end{aligned}\right\} \text {only if } \textit{max} = \textit{true}. \end{aligned}$$
  Then, for each individual *i*, the selection is done by
  34 $$\begin{aligned} \mathbf {p}_i^{(g+1)} = {\left\{ \begin{array}{ll} \mathbf {c}_i, &{} \text {if } v_i^{\mathrm{(C)}} < v_i^{(\mathrm{P})}.\\ \mathbf {p}_i^{(g)}, &{} \text {otherwise}. \end{array}\right. } \end{aligned}$$

In sum, after the initialization of the population (Step 1), the process updates the current population $\mathbf {p}$ consisting of $\hbox {NP}$ individuals and updates their fitness ${\mathbf {v}}^{(\mathrm{P})}$. This update loop (containing Step 2 and Step 3) is executed for a given number of generations $N_\mathrm{G}$.

Then, in the final step, the individual ${\mathbf {p}}_\mathrm{sol}$ is returned that has the optimal fitness value $v_\mathrm{opt}^{(\mathrm{P})}$. The value $v_\mathrm{opt}^{(\mathrm{P})}$ is either a minimum fitness in case of minimization, or a maximum fitness in case of maximization. Formally, this is done by setting

35 $$\begin{aligned} v_\mathrm{opt}^{(\mathrm{P})} = v_1^{(\mathrm{P})}. \end{aligned}$$

Then, for each individual *i*, do:

36 $$\begin{aligned} \left. \begin{aligned} v_\mathrm{opt}^{(\mathrm{P})} =\,&v_i^{(\mathrm{P})} \\ \hbox {sol} =\,&i \end{aligned}\right\} \text {only if } v_i^{(\mathrm{P})} < v_\mathrm{opt}^{(\mathrm{P})}. \end{aligned}$$

The corresponding optimal fitness value is returned depending on the boolean variable *max* specifying minimization or maximization, in our case maximization:

37 $$\begin{aligned} v_\mathrm{opt}^{(\mathrm{P})} \leftarrow -v_\mathrm{opt}^{(\mathrm{P})} \quad \text {only if } \textit{max} = \textit{true}. \end{aligned}$$

Algorithm output contains two parts: The first is ${\mathbf {h}}_\mathrm{sol} := {\mathbf {p}}_\mathrm{sol}$ containing the optimal MTRNN hyperparameters. The second is the corresponding fitness value $v_\mathrm{opt}^{(\mathrm{P})}$.

All these aforementioned Steps 1, 2, 3 are integrated in Algorithm 1 that realizes our proposed EO-MTRNN shown in Fig. 3.
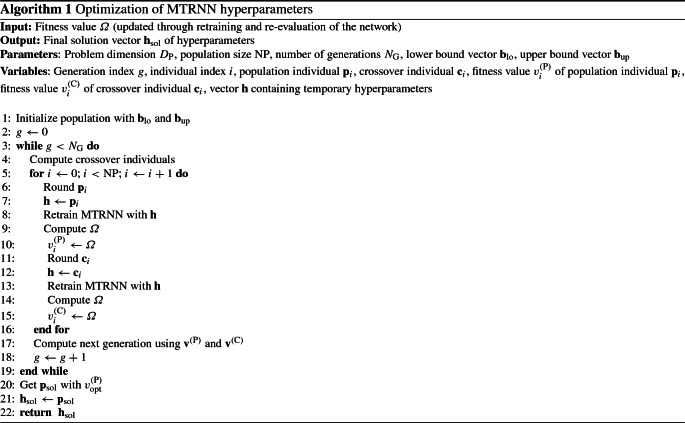


### Implementation

For the implementation of the EO-MTRNN, we did not use any frameworks or libraries. Our implementation was written in C++ and run on Linux (Ubuntu 16.04) on a conventional computer with an i7 central processing unit (CPU). Based on the mathematical and algorithmic descriptions in this paper, the EO-MTRNN can be implemented in any programming language of choice.

## Benchmark training dataset

Training the network on this benchmark dataset should yield an insight how the network performs depending on various factors such as the type of the training sequence, its dimension, and its length.

The benchmark training dataset is divided into sequences with one spatial dimension (elementary sequences) and sequences with multiple spatial dimensions. The multi-dimensional benchmark sequences are composed of the elementary sequences. Each spatial dimension of a benchmark sequence can be described mathematically, where *y* is the sample value and *t* is the discrete timestep. Gaussian noise $\eta$ was added with a mean $\mu = 0$ and variance $\sigma = 0.01$. This addition of noise simulates the property of teaching or training data, which would be collected in a real application scenario, for example when sensory-motor training data would be collected on a robot through kinesthetic teaching. The network should be robust to noise in the training data to a certain extent.

### One-dimensional sequences

**Fig. 4:**
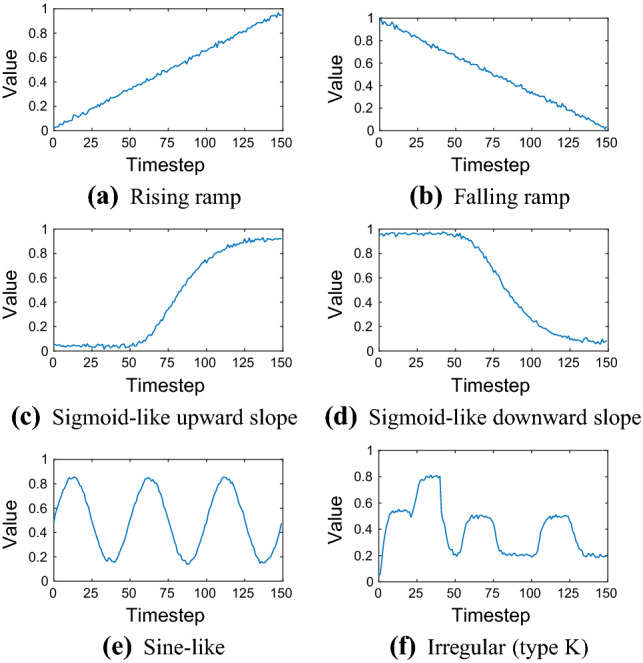
Elementary benchmark training sequences of length $L = 150$. See Table 2 for their mathematical description used to generate them

Here, we introduce training sequences with one spatial dimension only. They are also referred as elementary benchmark sequences. Table 2 provides the mathematical description to generate elementary benchmark sequences used to train and evaluate the network. The sequences with $L = 150$ are visualized in Fig. 4.

Note that for the sine-like sequence (Fig. 4e), we used its mathematical description in Table 2, incremented *t* by 0.02 to generate the function values, and assigned discrete timesteps to each of those values separately.

**Fig. 5:**
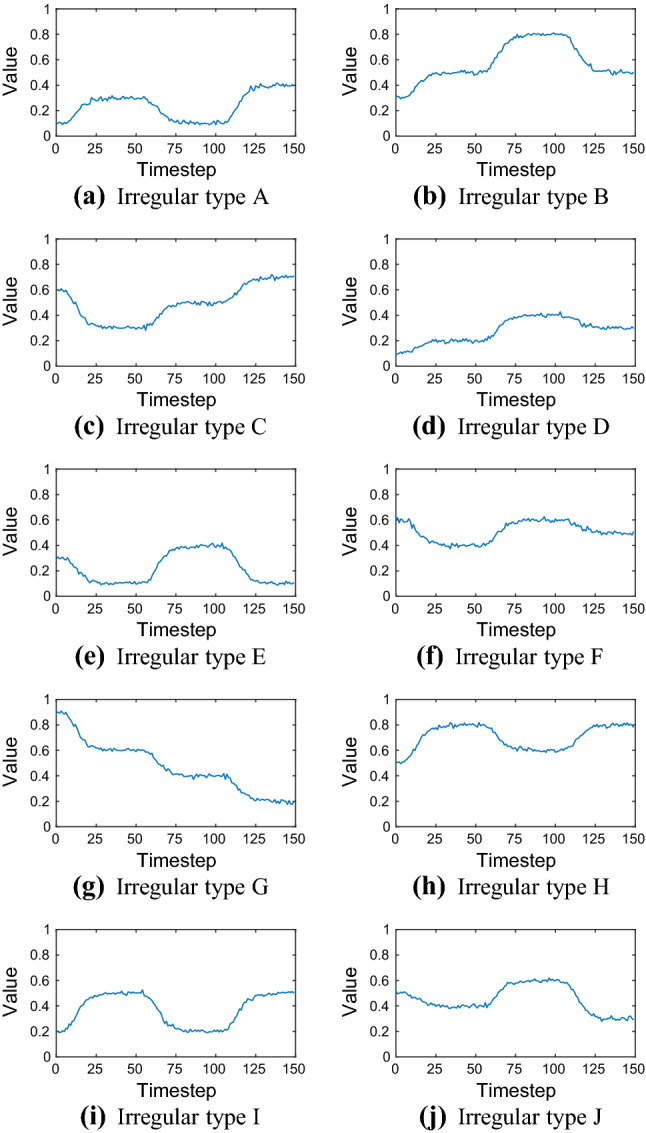
Elementary benchmark training sequences of irregular type. See Table 3 for their mathematical description used to generate them

Training data is often irregular in practice, i.e. it does not follow a regular shape like a ramp for example. Therefore, we added training sequences of irregular type to the benchmark data set. These irregular sequences can be mathematically approximated by Gompertz functions, see Table 3. The irregular benchmark sequences are shown in Fig. 5.

**Table 3:** Elementary benchmark training sequences of irregular type

Type	Length *L*	Interval	*a*	*d*	*e*
A	50	$0 \le t < 50$:	0.2	0	0.1
	100	$51 \le t < 100$:	$-\,0.2$	51	0.3
	150	$101 \le t < 150$:	0.3	101	0.1
B	50	$0 \le t < 50$:	0.2	0	0.3
	100	$51 \le t < 100$:	0.3	51	0.5
	150	$101 \le t < 150$:	$-\,0.3$	101	0.8
C	50	$0 \le t < 50$:	$-\,0.3$	0	0.6
	100	$51 \le t < 100$:	0.2	51	0.3
	150	$101 \le t < 150$:	0.2	101	0.5
D	50	$0 \le t < 50$:	0.1	0	0.1
	100	$51 \le t < 100$:	0.2	51	0.2
	150	$101 \le t < 150$:	$-\,0.1$	101	0.4
E	50	$0 \le t < 50$:	$-\,0.2$	0	0.3
	100	$51 \le t < 100$:	0.3	51	0.1
	150	$101 \le t < 150$:	$-\,0.3$	101	0.4
F	50	$0 \le t < 50$:	$-\,0.2$	0	0.6
	100	$51 \le t < 100$:	0.2	51	0.4
	150	$101 \le t < 150$:	$-\,0.1$	101	0.6
G	50	$0 \le t < 50$:	$-\,0.3$	0	0.9
	100	$51 \le t < 100$:	$-\,0.2$	51	0.6
	150	$101 \le t < 150$:	$-\,0.2$	101	0.4
H	50	$0 \le t < 50$:	0.3	0	0.5
	100	$51 \le t < 100$:	$-\,0.2$	51	0.8
	150	$101 \le t < 150$:	0.2	101	0.6
I	50	$0 \le t < 50$:	0.3	0	0.2
	100	$51 \le t < 100$:	$-\,0.3$	51	0.5
	150	$101 \le t < 150$:	0.3	101	0.2
J	50	$0 \le t < 50$:	$-\,0.1$	0	0.5
	100	$51 \le t < 100$:	0.2	51	0.4
	150	$101 \le t < 150$:	$-\,0.3$	101	0.6

Each sequence type can be described by $y(t) = a \exp (-b \exp (-c (t-d))) + e + \eta$, with $b = 10$, $c = 0.2$, and $\eta$ is Gaussian noise with constants $\mu = 0$ and $\sigma = 0.01$

### Multi-dimensional sequences

In practice, training sequences provided to a recurrent neural network typically have multiple spatial dimensions. For our benchmark data set, each spatial dimension contains one of the elementary benchmark training sequences from Table 3. The multi-dimensional benchmark sequences are described in Table 4. These sequences, especially the sine-like and irregular ones, often occur in robotics applications, e.g. when the robot is taught to manipulate objects (Jeong et al. 2012).

## Results

Using our proposed benchmark dataset, we conduct an empirical analysis of our proposed system. Section 5.1 deals with the network settings, as well as the termination criterion for optimizing the weights and initial potentials. In Sect. 5.2, we explain the metric we use to measure the learning capability. Sections 5.3 and 5.4 present the results of our proposed MTRNN when applied to sequence learning in different configurations, i.e. with and without pre- and postprocessing, and with and without early stopping, respectively. We then validate the correctness of our SA-DE implementation in Sect. 5.5. In Sect. 5.6, we add the SA-DE to the MTRNN and show how the automatic estimation of key network hyperparameters improves the learning ability. Finally, we also present examples of learning robot sensory-motor data in Sect. 5.7.

### Network settings and termination criterion of BPTT

For the learning of both, one-dimensional and multi-dimensional sequences (Sects. 4.1 and 4.2), we used the following parameterization in Table 5. Note that the variable number of IO neurons in Table 5 results from the given network configuration mode. For example, if the preprocessing is deactivated, a four-dimensional input pattern requires exactly four IO neurons, i.e. one-to-one mapping. The same pattern requires 20 IO neurons if preprocessing is activated (four-dimensional input times five-dimensional preprocessing weight vector). The numbers shown in Table 5 were kept fixed, until we proceeded with the hyperparameter estimation which we show later in this section. The values for the learning rates and momentum were kept fixed throughout all experiments; they were also kept fixed during the hyperparameter estimation that focused on the timescales and the number of neurons. The values for the learning rates and momentum are summarized in Table 6. For the optimization of weights and initial potentials, the BPTT terminated if the number of epochs reached 500,000 or if the training MSE reached $3.0 \times 10^{-5}$ or less, independent of the network configuration. In case of early stopping, the BPTT also stopped if the training MSE was below $9.0 \times 10^{-4}$ and the validation MSE started to rise.

**Table 4:** Multi-dimensional benchmark training sequences

Spatial dimensions	Elementary type
1	D
2	D, E
4	D, E, C, B
6	D, E, C, B, A, F
8	D, E, C, B, A, F, H, G
10	D, E, C, B, A, F, H, G, J, I

See Table 3 for each elementary type. For example, the two-dimensional sequence consists of type D as its first spatial dimension and type E as its second spatial dimension. Note that the order does not matter, and it was composed randomly

**Table 5:** Number of neurons and timescales for the learning

$N_\mathrm{IO}$	$N_\mathrm{FC}$	$N_\mathrm{SC}$	$\tau _\mathrm{IO}$	$\tau _\mathrm{FC}$	$\tau _\mathrm{SC}$
Variable	20	5	20	25	250

**Table 6:** Learning rates $\alpha$, $\beta _\mathrm{FC}$, $\beta _\mathrm{SC}$ and momentum $\eta$ kept fixed for all experiments

$\hbox {MSE}_{\mathrm{T}}$	$\alpha$	$\beta _\mathrm{FC}$	$\beta _\mathrm{SC}$	$\eta$
$\ge$ 0.03	0.6	0.6	0.6	0.9
$< 0.03$	0.4	0.4	0.4	0.9

### Metric for the learning capability

We investigate the learning capability depending on the length of the training sequences, the type of the training sequences, and their dimension. The learning capability is measured by $\varOmega$ defined in Eq. (27). In the following, we call this metric *R*-value, i.e. $R \in \mathbb {R}$ | $-1.0 \le R \le 1.0$, since it is a normalized sum of the correlation coefficients. Each correlation coefficient, given by Eq. (28), describes how well the prediction fits the observed data.

### Learning one-dimensional sequences

Table 7 compares four different configuration modes of running the network, and how the learning of sequences is affected by these modes.

**Table 7:** Learning of one-dimensional benchmark sequences

Length	Network configuration		Type of training sequence					
	Preprocessing	Early stopping	Ramp		Sigmoid-like		Sine	Irregular (type K)
			Falling	Rising	Downward slope	Upward slope		
50	*OFF*	*OFF*	0.992	$-$ 0.0415	0.997	0.998	0.937	0.479
50	*OFF*	*ON*	0.966	0.994	0.986	0.988	0.120	0.333
50	*ON*	*OFF*	0.998	0.998	0.995	0.996	0.945	0.318
50	*ON*	*ON*	0.965	0.970	0.987	0.996	0.900	0.501
100	*OFF*	*OFF*	0.994	$-$ 0.447	0.999	1.00	0.827	0.451
100	*OFF*	*ON*	0.953	0.982	0.943	0.944	0.285	0.0458
100	*ON*	*OFF*	0.996	0.999	0.998	0.999	0.644	0.410
100	*ON*	*ON*	0.940	0.954	0.941	0.962	0.491	0.419
150	*OFF*	*OFF*	0.995	$-$ 0.404	0.999	0.999	0.664	0.177
150	*OFF*	*ON*	0.930	0.950	0.901	0.905	$-$ 0.0408	0.106
150	*ON*	*OFF*	$-$ 0.617	0.999	0.999	0.999	0.196	0.793
150	*ON*	*ON*	0.707	0.844	0.885	0.939	0.172	0.488

The learning is measured by the *R*-value. Four different network configuration modes were compared, given the length of a training sequence

### Learning multi-dimensional sequences

**Fig. 6:**
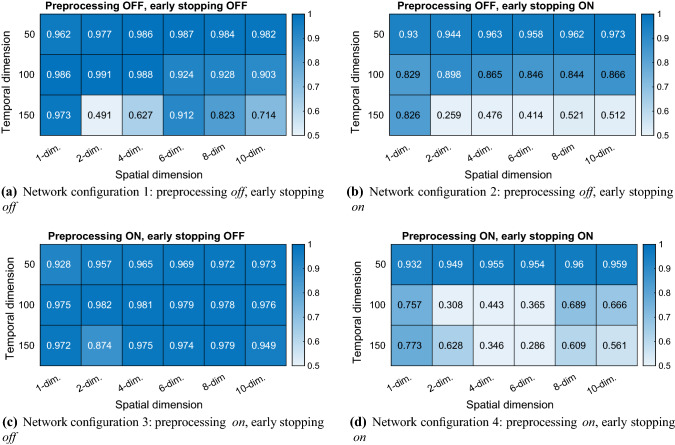
Learning of multi-dimensional benchmark training sequences. The learning is measured by the *R*-value indicated by the colour bar. Four different network configuration modes were compared (colour figure online)

**Fig. 7:**
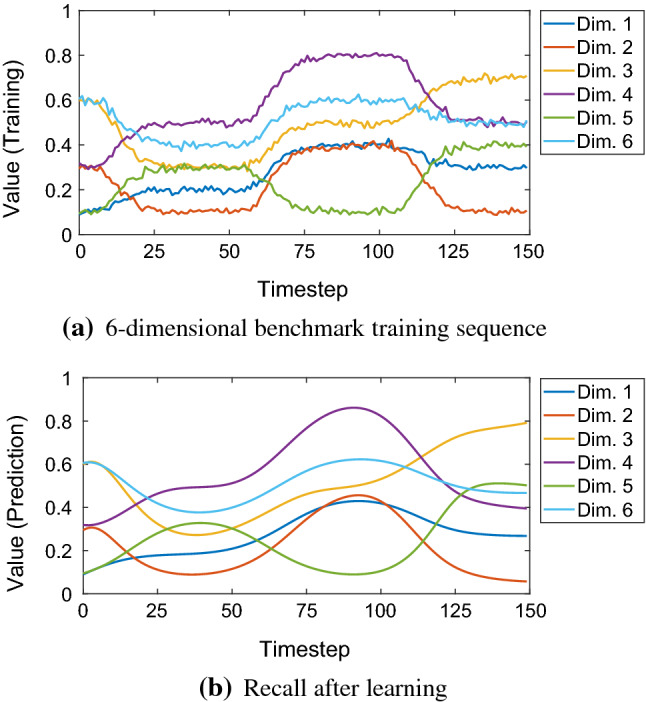
Learning and recall of the noisy six-dimensional benchmark training sequence with length $L = 150$. Network configuration: preprocessing *on*, early stopping *off*. Achieved *R*-value: 0.974. See Table 4 for details on the training data (colour figure online)

**Fig. 8:**
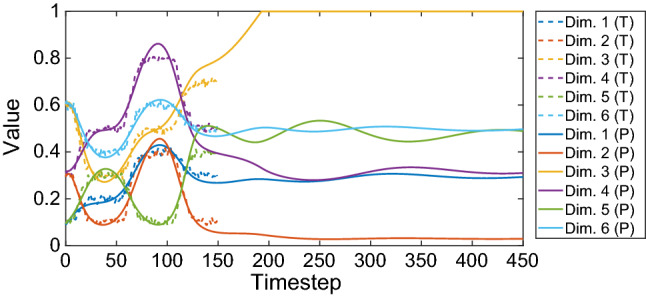
Recall with extrapolation from timestep 150 to 450. Dashed lines are the training sequence labelled (T), see also Fig. 7a. Solid lines are the predicted sequence labelled (P). The network tends to extrapolate the sequence based on the latest history of input–output activations, behaving like a type of predictive memory (colour figure online)

The network is trained with multi-dimensional sequences (Table 4). The learning results are summarized in Fig. 6. An example case of learning and recall is shown in Fig. 7. Figure 8 shows an example of the extrapolation behaviour of the net, i.e. the prediction of a sequence over a much longer timespan than the original timespan given in training.

### Validation of SA-DE implementation

In order to validate whether we correctly implemented the SA-DE, we compared our numerical results against the results obtained from the original version in Brest et al. (2006), with the same parameter settings. Table 8 shows that our results concur with Brest et al. (2006); hence, our implementation is correct. In particular, the results on benchmark functions $f_8$, $f_9$, $f_{10}$, $f_{11}$, $f_{12}$, $f_{13}$ are important to consider, since they demonstrate the ability of the method to find a global optimum despite a high number of local optima (Brest et al. 2006; Yao et al. 1999; Törn and Žilinskas 1989). Note that this validation only serves to ensure a correct implementation of the SA-DE optimization method.

**Table 8:** Comparison of numerical results using the benchmark function set *F* from Brest et al. (2006)

*F*	# Gen.	SA-DE (own)	SA-DE (Brest et al. 2006)
$f_1$	1500	$2.0 \times 10^{-16}$	$1.1 \times 10^{-28}$
	2400	$5.7 \times 10^{-29}$	
$f_2$	2000	$3.3 \times 10^{-14}$	$1.0 \times 10^{-23}$
	3200	$1.5 \times 10^{-23}$	
$f_3$	5000	$4.9 \times 10^{-3}$	$3.1 \times 10^{-14}$
	13, 000	$2.4 \times 10^{-14}$	
$f_4$	5000	$2.2 \times 10^{-9}$	0
$f_5$	20, 000	$2.9 \times 10^{-30}$	0
$f_6$	1500	0	0
$f_7$	3000	$2.83 \times 10^{-1}$	$3.15 \times 10^{-3}$
$f_8$	9000	$-\,12{,}569.5$	$-\,12{,}569.5$
$f_9$	5000	0	0
$f_{10}$	1500	$3.7 \times 10^{-9}$	$7.7 \times 10^{-15}$
	2500	$7.2 \times 10^{-15}$	
$f_{11}$	2000	0	0
$f_{12}$	1500	$1.7 \times 10^{-17}$	$6.6 \times 10^{-30}$
	2400	$6.4 \times 10^{-30}$	
$f_{13}$	1500	$1.1 \times 10^{-16}$	$5.0 \times 10^{-29}$
	2400	$5.3 \times 10^{-29}$	
$f_{16}$	100	$-\,1.03163$	$-\,1.03163$
$f_{17}$	100	0.397887	0.397887
$f_{18}$	100	3	3

The main results are the minima (columns 3 and 4) of particular benchmark functions; the minima are averaged over 50 independent runs. The only purpose of this comparison is to validate a correct implementation of the SA-DE method. Since our results concur with Brest et al. (2006), our implementation of the SA-DE method is correct

### Improvement of learning capability by evolutionary optimization

Here, we investigated whether our proposed Algorithm 1 can improve the learning of given training data. Algorithm 1 performs the autonomous hyperparameter estimation (AHE), in order to yield an evolutionary optimized network. We set the problem dimension (see Table 1) to five, i.e. our optimization system suggested the number of context neurons $N_\mathrm{FC}$, $N_\mathrm{SC}$, and the different timescales $\tau _\mathrm{IO}$, $\tau _\mathrm{FC}$, $\tau _\mathrm{SC}$. For the SA-DE, we set the population size $\hbox {NP} = 4$ (minimum number possible due to the property of SA-DE) and the number of generations $N_\mathrm{Gen.} = 10$. In contrast to the original DE, we do not need to care about the values for the crossover probability CR and the differential weight *F*, since they are autonomously adjusted through the self-adapting property of SA-DE. The values of *F* and CR were updated by Eqs. (29) and (30), respectively, with $F \in [0.1, 1.0]$ and $CR \in [0.0, 1.0]$. Regarding the search space, we set the bounds for the context neurons to $5 \le N \le 30$ for fast context and slow context, respectively. We set the bounds for the timescales to $2 \le \tau \le 300$ for input–output, fast context, and slow context group, respectively.

For comparison purposes, we used a weak to reasonable default parameterization of the network, see Table 9.

**Table 9:** Default parameterization for comparison purpose

$N_\mathrm{IO}$	$N_\mathrm{FC}$	$N_\mathrm{SC}$	$\tau _\mathrm{IO}$	$\tau _\mathrm{FC}$	$\tau _\mathrm{SC}$
variable	15	5	10	20	40

**Fig. 9:**
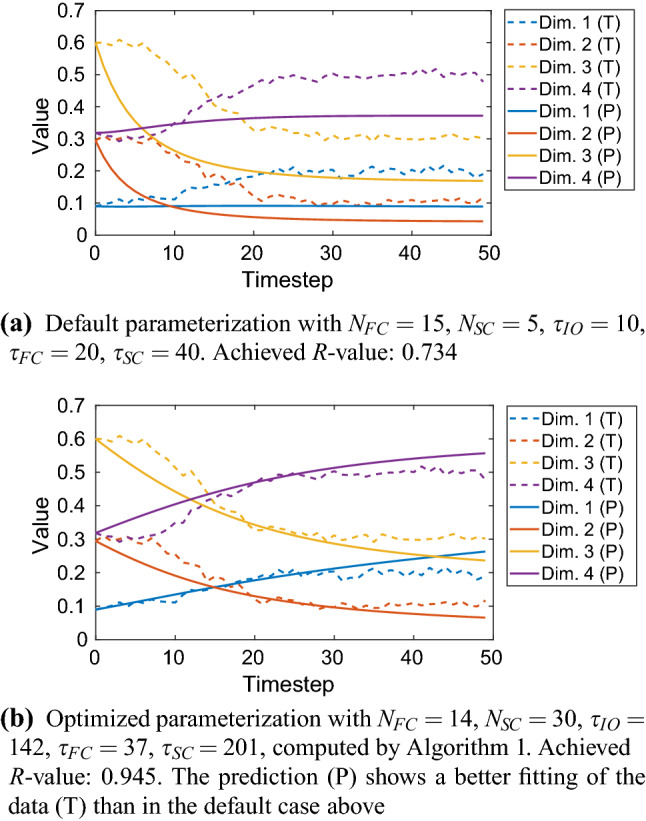
Example of hyperparameter estimation: The target sequence (dashed lines) had 4 spatial dimensions and length 50. The configuration was preprocessing *on* and early stopping *on* meaning that only 33 of 50 samples were used for training. The recall performance of a weak to reasonable default parameterization is shown in **a**. Applying our proposed hyperparameter optimization to $N_\mathrm{FC}$, $N_\mathrm{SC}$, $\tau _\mathrm{IO}$, $\tau _\mathrm{FC}$, $\tau _\mathrm{SC}$ increased the performance by 28.7 % without overfitting the data, see **b** (colour figure online)

#### Single sequences

**Fig. 10:**
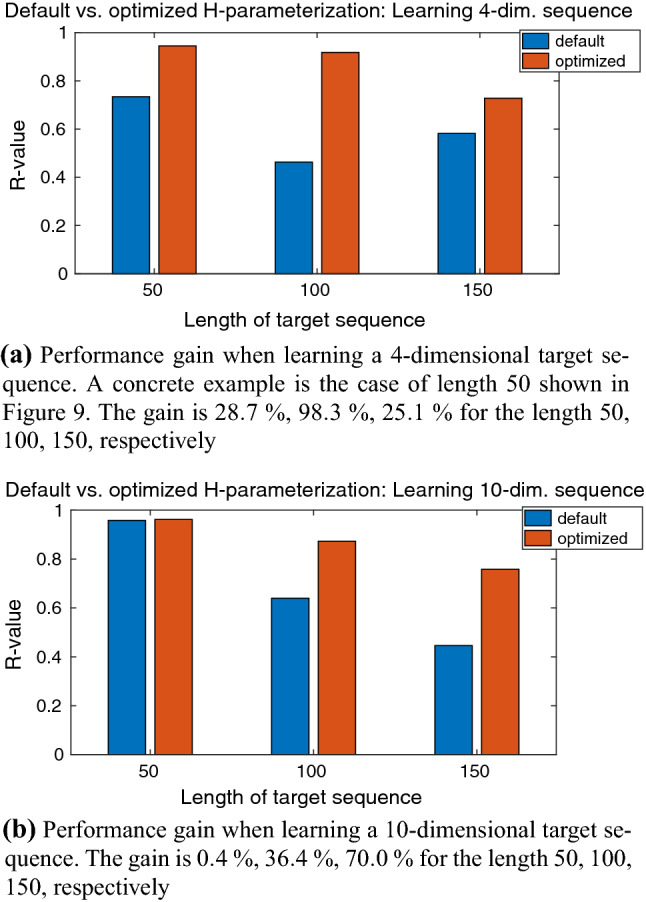
Default versus optimized hyperparameterization: Comparison of learning performance when learning a multi-dimensional target sequence with different lengths; network configuration was preprocessing *on* and early stopping *on*. From these cases, it follows an average performance gain of approximately 43 % compared to the default parameterization given in Table 9 (colour figure online)

The number of IO neurons was predetermined by the given network configuration; it was the same as described in Sect. 5.1. Figure 9 shows an example where the hyperparameter estimation improved the learning result. To summarize, we show a performance comparison between a default and an optimized hyperparameterization in Fig. 10.

#### Multiple sequences

Using our benchmark sequences of irregular type (Fig. 5), we also evaluate different cases of *simultaneous* learning of multiple sequences. We simultaneously trained 7 sequences; each of them had 4 spatial dimensions. The assembly of these sequences is given in Table 10.

**Table 10:** Benchmark training sequences of irregular type; all 7 sequences were trained simultaneously

Sequence number	Spatial composition
1	D, E, C, B
2	A, F, H, G
3	J, I, D, E
4	B, C, E, D
5	E, A, J, B
6	I, J, H, A
7	F, G, D, C

Each spatial dimension contains an elementary sequence of irregular type (see Fig. 5 for the visualization of each spatial dimension). The arrangement of the spatial dimensions does not matter; it was composed randomly

We investigated three different cases of temporal dimensions, i.e. sequence lengths: $L = 50$, $L = 100$, and $L = 150$. The optimized hyperparameters were computed by Algorithm 1 trained with 7 sequences simultaneously, each with $L = 150$. The obtained hyperparameters were then kept constant and used to train the network with different lengths per sequence: $L = 50$, $L = 100$, $L = 150$. Since 7 sequences were trained simultaneously for each case, the total number of samples was 350, 700, and 1050, respectively. Each case was also trained with default hyperparameters (Table 9) to compare the performance.

We used an 11-dimensional weight vector for our analytical pre- and postprocessor generating sparse representation of IO data. The middle element of this vector is close to 1.0, while the other elements are close to 0.0. Beginning with the first and going to the last, the elements of the weight vector $\mathbf {v}^T$ were 0.007812, 0.015625, 0.03125, 0.0625, 0.125, 0.99, 0.125, 0.0625, 0.03125, 0.015625, and 0.007812.

**Fig. 11:**
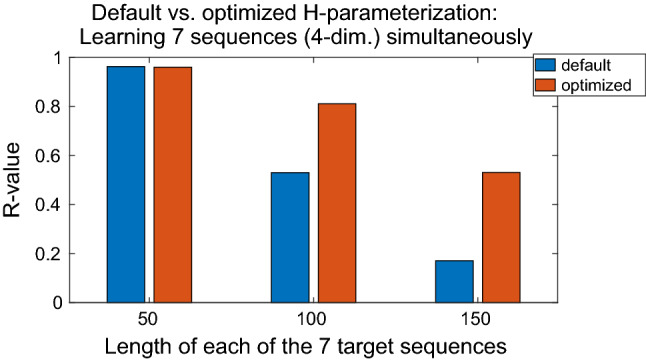
Default versus optimized hyperparameterization for multiple sequences trained simultaneously: Network configuration was preprocessing *on* and early stopping *off*. The greater the size of training data, the more the evolutionary optimization is worth it. Cases $L = 100$ and $L = 150$ have great differences in the *R*-value, respectively: 0.530 versus 0.811 (53.0 % gain) and 0.171 versus 0.531 (210.5 % gain, albeit poor performance in the default case). As an example, the optimized case with $L = 100$ is visualized by Figs. 12 and 13 (colour figure online)

The computed hyperparameters were $N_\mathrm{FC} = 10$, $N_\mathrm{SC} = 9$, $\tau _\mathrm{IO} = 19$, $\tau _\mathrm{FC} = 167$, $\tau _\mathrm{SC} = 37$. Looking at the timescales, the roles of the fast context and slow context neurons were swapped by the evolutionary algorithm, i.e. the fast context group became slow context and vice versa. Nevertheless, this can be justified by the results when comparing the learning performance with the default parameterization. The performance results are summarized in Fig. 11.

**Fig. 12:**
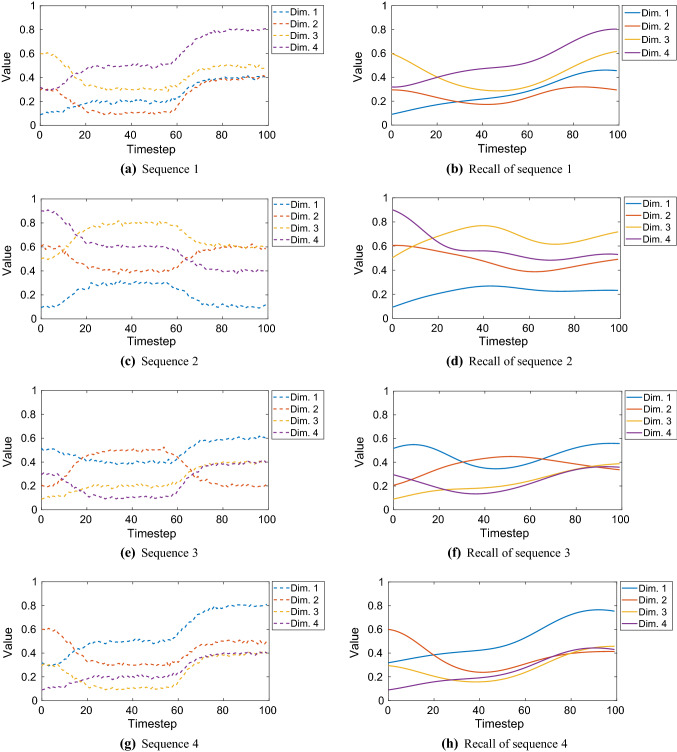
Simultaneous training of 7 benchmark sequences ($L = 100$) and their recall: sequences 1–4. See Fig. 13 for the sequences 5 to 7 (colour figure online)

**Fig. 13:**
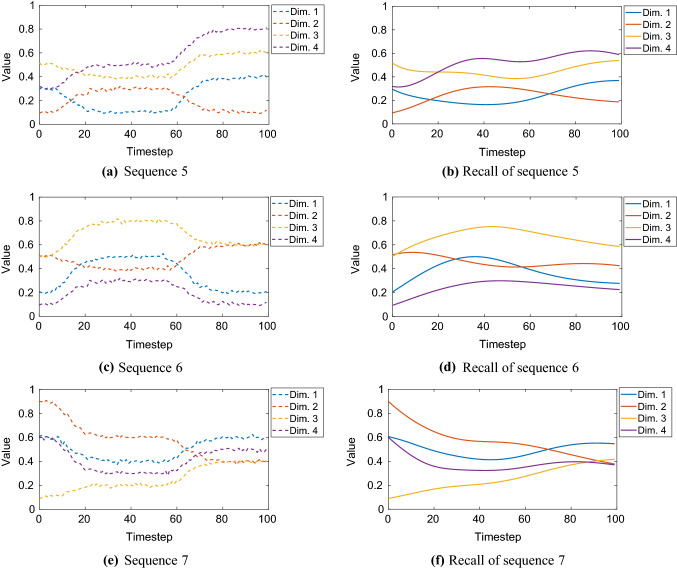
Simultaneous training of 7 benchmark sequences ($L = 100$) and their recall: sequences 5–7 (colour figure online)

**Fig. 14:**
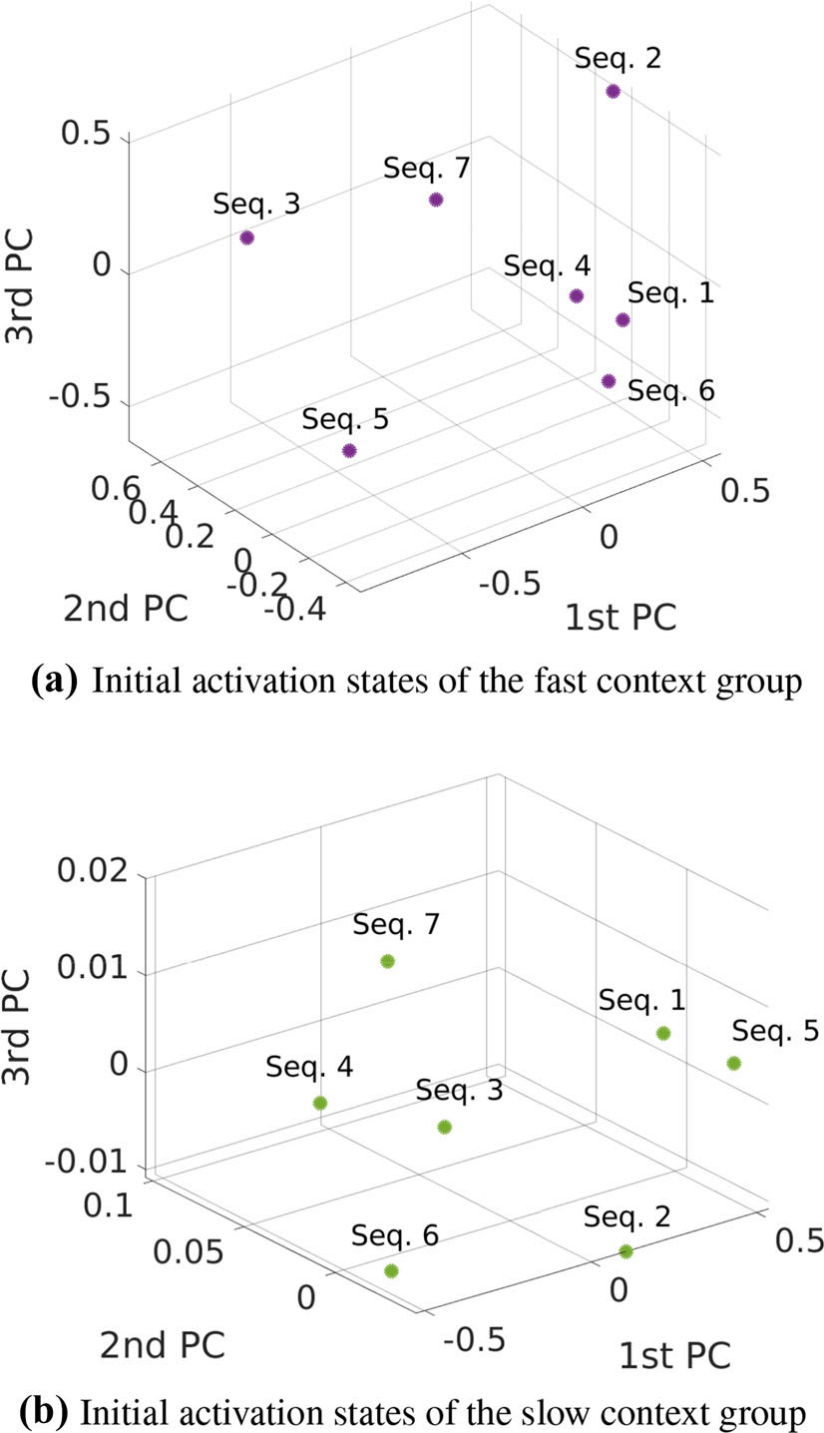
Initial activation states of the context group in principal component (PC) space after being trained with 7 sequences simultaneously (each sequence with $L = 100$), using optimized hyperparameters. It can be seen that all 7 sequences are clearly separable in the activation space of each context group. Mapped from each initial activation state of fast and slow context, the corresponding sequence can be recalled, see Figs. 12 and 13, by using the learned weights

For each case of training with default and optimized hyperparameters, the number of epochs was $10^6$. An example of sequence recall is shown in Figs. 12 and 13, where 7 sequences (each $L = 100$) were trained simultaneously with the optimized hyperparameters. Corresponding to this example, we also investigated the self-organization of the initial activation states of the context neurons. Figure 14 shows these initial activation states.

In order to find suitable hyperparameters for these benchmark sequences trained simultaneously, the number of epochs was set to 15, 000. This is relatively short, but was necessary due to time restrictions. Using 15, 000 epochs per single individual of the network population going through the evolutionary process and using the learning rates in Table 6, Algorithm 1 takes roughly 35 h to find suitable hyperparameters for 1050 (i.e. $7 \times 150$) sample vectors with 4 dimensions, when run on an i7-7500U CPU (2.7 Ghz). Note that this can be significantly speeded up by running the proposed algorithm on a graphics processing unit (GPU) supporting parallel programming, e.g. CUDA. This is important to consider because many more epochs per training and more evolutionary generations can be computed within the same timeframe. Consequently, GPU usage would boost the learning performance within the same timeframe.

### Application to sensory-motor data from robots

In this section, we investigated our network performance when trained with action sequences that were shown to a Sony QRIO humanoid robot in Yamashita and Tani (2008). In their paper, the robot was fixed to a stand and manipulated a cubic object that was placed on a workbench in front of the robot. The action sequences taught to the robot consisted of several manipulation primitives using both arms, e.g. reach and grasp the object, and move the object up and down three times. Each action sequence begins and ends in a defined home position. The action sequences are sensory-motor sequences; each of them has 10 spatial dimensions but a different length depending on the interaction taught. Among the 10 spatial dimensions, the first 8 dimensions represent the DOF of the robot arms: 3 DOF shoulder and 1 DOF elbow per arm. A 2 DOF head-neck joint followed the object automatically by a given visual servoing mechanism. These 2 dimensions represent the visual input (horizontal *X*, vertical *Y*), describing the object position relative to the robot’s visual field. Each 10-dimensional sensory-motor pattern was sampled every 150 ms.

In the following two paragraphs, we adopt parts of the training data from Yamashita and Tani (2008) and apply it to our proposed network. The values for the learning rates and momentum were the same as in Table 6. For our analytical pre- and postprocessor generating sparse representation of IO data, we used the same 11-dimensional weight vector as in Sect. 5.6.2. With eight-dimensional proprioceptive and two-dimensional visual data, this resulted in 88 neurons encoding the motor part of the IO group and 22 neurons encoding the visual part of the IO group. This yielded $N_\mathrm{IO} = 110$. If the IO group is split in two parts, e.g. proprioceptive and visual, the connective weights between theses two parts are zero.

**Fig. 15:**
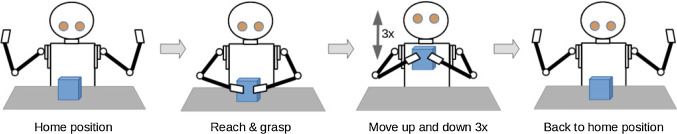
Robot task in Yamashita and Tani (2008) to obtain sensory-motor data through kinesthetic teaching. In home position, the robot is facing a box (blue) on a workbench (grey). It reaches and grasps the box. Then, the robot moves the box up and down three times, with its head cameras always focusing on the box by moving the head-neck joint accordingly. Finally, the robot returns back to home position (colour figure online)

**Fig. 16:**
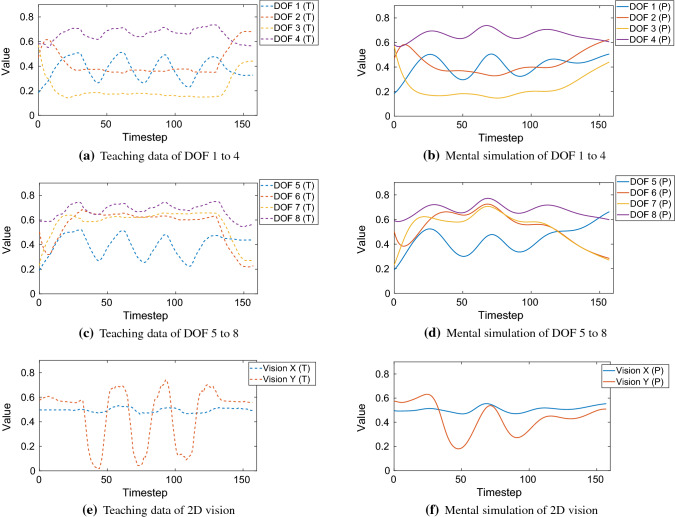
Left side: Teaching data of the behaviour sequence consisting of reaching, grasping, and up-down behaviour in Yamashita and Tani (2008). This behaviour sequence is visualized in Fig. 15. Right side: Recall of the sequence (i.e. mental simulation) by our EO-MTRNN, although with default (non-optimized) hyperparameters reflecting a worst-case scenario. The EO-MTRNN is still able to sufficiently learn the sequence despite an adverse choice of hyperparameters. Note that the hyperparameters are *not* optimized in this case; here, it was of interest whether the proposed network can still preserve the task structure in case of a single learning procedure *without* going through the evolutionary optimization process (colour figure online)

**Learning sensory-motor data with adverse hyperparameters** In the following experiment, the objective is to investigate the mental simulation performance of our EO-MTRNN when deliberately operated with hyperparameters that are far from the optimum. We selected the sequence representing the following behaviour: starting from home position, reach and grasp the object (a box) with both arms, and move it up and down three times, then go back to home position. This robot task is visualized in Fig. 15 and taught to the robot through kinesthetic teaching. Kinesthetic teaching, in which a human takes the arms of the robot and shows it the task, is used to collect the sensory-motor data. This sensory-motor data are used as training data for the MTRNN. The data encoding this task is also shown in the first column (Teach) in Yamashita and Tani (2008, p. 7, Fig. 4). We adopted these data and trained our EO-MTRNN with this sequence, however, using a default configuration *without* hyperparameter estimation, in order to investigate a worst-case scenario. We set the number of context neurons and the values of timescales as follows: $N_\mathrm{FC} = 15$, $N_\mathrm{SC} = 5$, $\tau _\mathrm{IO} = 10$, $\tau _\mathrm{FC} = 20$, $\tau _\mathrm{SC} = 40$. Our network reached an MSE of $3 \times 10^{-6}$ after 821,990 epochs. We validated the recall of this sequence, i.e. its mental simulation. Figure 16 shows the results. The results show a reasonable performance in recalling or predicting the sequence; the *R*-value was 0.550.

**Fig. 17:**
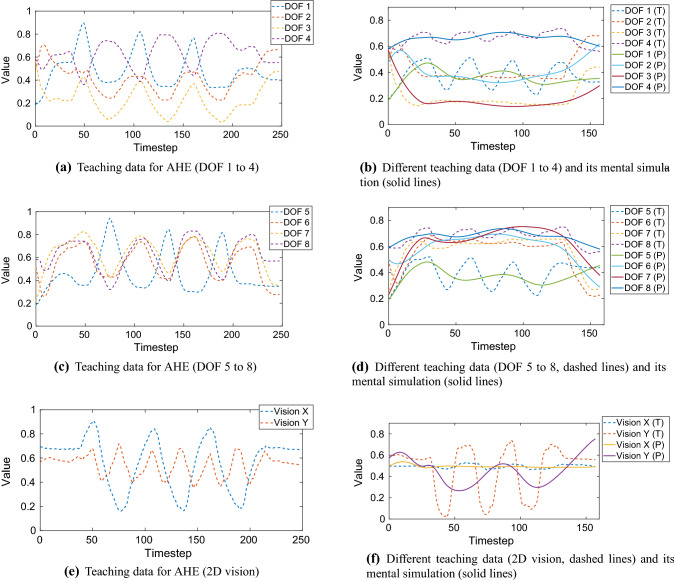
Left side **a**, **c**, **e** Teaching data of the behaviour sequence used for autonomous hyperparameter estimation (AHE). It encodes reaching and grasping the box, and moving it left and right three times. Right side **b**, **d**, **f** Dashed lines are the teaching data of the task shown to the robot in Fig. 15. These data are different from the data on the left used for AHE. For example, the data on the left side encode moving the box left to right, instead of up and down. The solid lines are the mental simulation of the robot task by the EO-MTRNN. These results show *generalization ability*: The EO-MTRNN is able to sufficiently learn the task (reach and grasp the box, move it *up and down* three times), although it estimated its hyperparameters for another task (reach and grasp the box, move it *left and right* three times). The approximation can be further improved by increasing the number of generations ($N_\mathrm{Gen.} > 10$) or by increasing the number epochs per individual of the network population (colour figure online)

**Generalization ability of autonomous hyperparameter estimation** The objective is to investigate the generalization ability of the EO-MTRNN. We evaluate whether the hyperparameters that were obtained based on a particular teaching data would still enable our EO-MTRNN to learn different data. By different data, we mean data that was *not* part of the teaching data used for the optimization of hyperparameters. For this purpose, we selected the longest sequence that was taught in the experiment by Yamashita and Tani, and we used it as input for the optimization of hyperparameters. The sequence encodes robot actions similar to, but not the same as the one depicted in Fig. 15. For example, the box is moved left to right, instead of up and down. We used this sequence as teaching data for the AHE. Then, we used the automatically estimated hyperparameters to train our network with the former behaviour sequence consisting of reaching, grasping, and moving up and down (Fig. 15). In sum, the obtained hyperparameters were used to learn a *different* sensory-motor sequence, i.e. not encountered during the AHE process. Teaching data and results of recall are shown in Fig. 17. The AHE process yielded $N_\mathrm{FC} = 30$, $N_\mathrm{SC} = 18$, $\tau _\mathrm{IO} = 64$, $\tau _\mathrm{FC} = 57$, $\tau _\mathrm{SC} = 290$. For training the different sequence, that is not seen during AHE process, we set the target MSE to $9.0 \times 10^{-6}$ that was reached after $10^6$ epochs. The achieved *R*-value was 0.622.

## Discussion

### Configurations of the proposed MTRNN

We designed our benchmark training dataset to capture a wide variety of possible sequences which a MTRNN could possibly encounter. The step-wise increment of the spatial dimensions of the benchmark sequences, e.g. from 1 to 2 to 4 etc., along with the different temporal lengths, should help to investigate how the learning ability of the network scales with the increase in spatial and temporal dimensions. Gaussian noise was added to the training data in order to simulate real application scenarios, for example, when the network is applied to learn sequences from noisy sensory-motor data collected on a robot.

We started our evaluation of the learning ability by comparing four different network configuration modes that are combinations of preprocessing (*PP*) and early stopping (*ES*). The schema for preprocessing was entirely analytical, i.e. without any auxiliary neural networks.

For the sequence learning analysis, summarized by Table 7 and Fig. 6, we chose a hyperparameterization similar to the descriptions in Yamashita and Tani (2008), with a careful focus on the ratio between the timescales $\tau _\mathrm{FC}$ and $\tau _\mathrm{SC}$.

Table 7 shows that a preprocessing of the input dimension increases the learning capability. A combination of active preprocessing and early stopping (i.e. *PPon* and *ESon*) is beneficial compared to configurations in which one or both of them are disabled.

For the learning of multi-dimensional sequences (Fig. 6), the combination of an active preprocessing and deactivated early stopping (*PP on* and *ES off*) shows the best results. In addition, we observed that preprocessing speeds up the BPTT convergence up to ten times, compared to configurations without preprocessing.

We used sigmoid activation for all units of the networks. Using other activation functions may influence the results, but that is beyond the scope of this work.

### Evolutionary optimized MTRNN

We validated our implementation of SA-DE and our results concurred with Brest et al. (2006). This shows the ability of SA-DE to escape from a high number of local optima and to locate the global optimum.

The results in Figs. 9 and 10 demonstrate that our proposed evolutionary optimization system can improve the learning capability of the MTRNN. The average improvement of 43% for the training of single sequences was computed from the different evaluation cases shown in Fig. 10. We decided to optimize the network configuration with active preprocessing and early stopping, i.e. *PPon* and *ESon*, since this configuration yielded some weaknesses in learning multi-dimensional sequences with a length greater than 100 (see Fig. 6d).

For the training of multiple sequences simultaneously, the evolutionary optimization of hyperparameters turned out to be beneficial particularly for learning sequences with increasing length, e.g. $L \ge 100$. An adverse (manual) choice of hyperparameters significantly deteriorates the learning performance, as was seen in the default cases in Fig. 11. There, evolutionary optimization yielded a performance gain of up to 131%.

Due to computational limitations, i.e. all experiments were conducted using a conventional CPU, we decided to use a minimal optimization setting by $\hbox {NP} = 4$ and $N_\mathrm{Gen.} = 10$. This yielded a minimal optimization, since the setting in many benchmark problems is $\hbox {NP} = 100$ and $N_\mathrm{Gen.} \ge 100$, like in Brest et al. (2006). Since we ran our optimization on CPU, this minimal setting with 4 individuals and 10 generations, including the retraining of the network, took several hours to complete. Nevertheless, the optimization setting $\hbox {NP} = 4$ and $N_\mathrm{Gen.} = 10$ already improved the learning capability for the given network configuration. In most of the conducted experiments, our proposed hyperparameter estimation system delivered similar ratios of $\tau _\mathrm{FC}$ and $\tau _\mathrm{SC}$ as suggested in the literature (Sect. 2.1). It also showed that the number of context neurons has less impact on the learning performance than the timescales have.

### Application in robotics

Section 5.7 shows the performance of our EO-MTRNN on data that were collected on a humanoid robot in Yamashita and Tani (2008). We validated training and recall of an action sequence by our proposed network in a non-optimized configuration, where we deliberately chose an adverse parameterization to investigate whether it is still able to sufficiently learn the data (Fig. 16). Compared to a very good sequence recall in Yamashita and Tani (2008) using optimal values for timescales and context neurons, the recall performance of the proposed EO-MTRNN was quite reasonable, although we switched off the evolutionary optimization in order to investigate a non-optimal ratio of the timescales ($\tau _\mathrm{IO} = 10$, $\tau _\mathrm{FC} = 20$, $\tau _\mathrm{SC} = 40$) and used relative few context neurons ($N_\mathrm{FC} = 15$, $N_\mathrm{SC} = 5$).

Then, we switched on the evolutionary optimization mechanism and validated the generalization ability of the proposed network, i.e. how well the network can learn and recall data that were *not* part of the hyperparameter optimization process (Fig. 17). For a good generalization ability, the hyperparameter optimization should be based on a rich variety of data. This is why we chose an action sequence containing oscillatory patterns covering a wide value range.

The EO-MTRNN allows an easier application in sensory-motor processing. Compared to Yamashita and Tani (2008) where two TPM networks needed to be trained in addition to the MTRNN before the robot could be controlled, our EO-MTRNN offers the possibility to directly feed the sensory-motor samples into the network, i.e. without the necessity to train auxiliary networks for pre- and postprocessing. The EO-MTRNN allows an one-to-one mapping where each element of a sensory or motor vector is directly mapped to one IO neuron activation, for example in Wieser and Cheng (2014), Wieser and Cheng (2016) and Burger et al. (2017).

Moreover, the results have shown that the EO-MTRNN can learn from a minimum amount of samples, also investigated in Wieser and Cheng (2014) and Wieser and Cheng (2016). This is a useful and necessary ability for developmental robots that robustly bootstrap their sensory-motor skills from limited amount of data over a continuum of developmental stages (Wieser and Cheng 2018).

In the past, the hyperparameterization of the network had to be manually estimated dependent on the sensory-motor data that the robot collected. Now, the evolutionary optimization mechanism allows to automatically reconfigure the network over time, e.g. when the robot samples a new set of data that needs to be learned. It allows a form of self-organization in terms of restructuring itself to better fit newly collected data. This means a higher autonomy of the robot, since a human does not have to stop the system to change the hyperparameters in an attempt to better learn newly collected data.

Note that we did not conduct a training of multiple *robotic* sequences simultaneously due to time restrictions. As we explained in Sect. 5.6.2, our study of simultaneous multiple sequence training took several days on a conventional computer with CPU (3 cases of different length shown in Fig. 11, evolutionary optimization for the third case of 1050 sample vectors took 35 h). We can conclude that our proposed model can only scale up its performance to larger sets of data, e.g. on robots, if the algorithm is implemented on parallel processing hardware such as GPU. This means that any limitation of performance is due to the hardware our algorithm runs on.

In Wieser and Cheng (2014), Wieser and Cheng (2016), Wieser and Cheng (2018) and Burger et al. (2017), the EO-MTRNN is trained with multiple robotic sequences simultaneously (without optimization of hyperparameters), and those sequences are collected by the robot’s autonomous exploration of degrees of freedom and successive interaction phase. The action selection system proposed in Wieser and Cheng (2014) and Wieser and Cheng (2016) switches between these multiple sequences to generate meaningful robot behaviour, running on conventional hardware, i.e. CPU.

## Conclusion

We proposed to model the neural plasticity of the cortex by an EO-MTRNN. When training data significantly changes over time, that is likely to be the case over multiple developmental stages of artificial agents, the EO-MTRNN has the ability to estimate all its neural timescales and to restructure itself by using evolutionary optimization in combination with BPTT. For a mixture of different sequences, ranging from 4 to 10 spatial dimensions and 50 to 150 temporal dimensions, this yields roughly 43% better approximation performance than solely optimizing a given number of synaptic weights and initial potentials. Our study of training multiple sequences simultaneously confirmed this performance gain and showed that the learned sequences can be clearly separated in the context memory of the network. Moreover, the evolutionary optimization yields a higher autonomy, in particular when the EO-MTRNN controls a robot over long term, since human intervention for stopping and reconfiguring the system can be reduced.

A next step would be to transfer the proposed system to GPU-based computing that would significantly reduce the time taken for the optimization procedures, thus making it possible to learn much greater amount of data, and to further improve the quality of learning since more network generations can be computed.

## Footnotes

1 Meta-parameters are also termed control parameters; they are the parameters of an optimization method which in turn is used to optimize the parameters of a target system.

## References

[CR1] Alnajjar F, Yamashita Y, Tani J (2013). The hierarchical and functional connectivity of higher-order cognitive mechanisms: neurorobotic model to investigate the stability and flexibility of working memory. Front Neurorobot.

[CR2] Anderson RL (1953). Recent advances in finding best operating conditions. J Am Stat Assoc.

[CR3] Arie H, Arakaki T, Sugano S, Tani J (2012). Imitating others by composition of primitive actions: a neuro-dynamic model. Robot Auton Syst.

[CR4] Badre D, D’Esposito M (2009). Is the rostro-caudal axis of the frontal lobe hierarchical?. Nat Rev Neurosci.

[CR5] Barron AR (1993). Universal approximation bounds for superpositions of a sigmoidal function. IEEE Trans Inf Theory.

[CR6] Bergstra J, Bengio Y (2012). Random search for hyper-parameter optimization. J Mach Learn Res.

[CR7] Brest J, Greiner S, Bošković B, Mernik M, Žumer V (2006). Self-adapting control parameters in differential evolution: a comparative study on numerical benchmark problems. IEEE Trans Evol Comput.

[CR8] Burger W, Wieser E, Dean-Leon E, Cheng G (2017) A scalable method for multi-stage developmental learning for reaching. In: Proceedings of the IEEE international conference on development and learning and epigenetic robotics, pp 60–65

[CR9] Cybenko G (1989). Approximation by superpositions of a sigmoidal function. Math Control Signals Syst.

[CR10] Eberhart R, Kennedy J (1995) A new optimizer using particle swarm theory. In: Proceedings of the IEEE sixth international symposium on micro machine and human science, pp 39–43

[CR11] Elman JL (1990). Finding structure in time. Cogn Sci.

[CR12] Friston K (2008). Hierarchical models in the brain. PLoS Comput Biol.

[CR13] Friston K (2010). The free-energy principle: a unified brain theory?. Nat Rev Neurosci.

[CR14] Fuster JM (2001). The prefrontal cortex—an update: time is of the essence. Neuron.

[CR15] Goodfellow I, Bengio Y, Courville A (2016). Deep learning.

[CR16] Huttenlocher P (1990). Morphometric study of human cerebral cortex development. Neuropsychologia.

[CR17] Huttenlocher P, Dabholkar A (1997). Regional differences in synaptogenesis in human cerebral cortex. J Comp Neurol.

[CR18] Jeong S, Arie H, Lee M, Tani J (2012). Neuro-robotics study on integrative learning of proactive visual attention and motor behaviors. Cogn Neurodyn.

[CR19] Johnson MH, Bremner JG, Wachs TD (2010). Functional brain development during infancy. The Wiley-Blackwell handbook of infant development.

[CR20] Johnson MH, de Haan M (2015). Developmental cognitive neuroscience.

[CR21] Jordan MI (1997). Serial order: a parallel distributed processing approach. Adv Psychol.

[CR22] Jung M, Hwang J, Tani J (2015). Self-organization of spatio-temporal hierarchy via learning of dynamic visual image patterns on action sequences. PLoS ONE.

[CR23] Kennedy J, Eberhart R (1995) Particle swarm optimization. In: Proceedings of the IEEE international conference on neural networks, pp 1942–1948

[CR24] Kiebel SJ, Daunizeau J, Friston KJ (2008). A hierarchy of time-scales and the brain. PLoS Comput Biol.

[CR25] Kohonen T (1982). Self-organized formation of topologically correct feature maps. Biol Cybern.

[CR26] Lee CY, Yao X (2004). Evolutionary programming using mutations based on the Lévy probability distribution. IEEE Trans Evol Comput.

[CR27] Liu J, Lampinen J (2005). A fuzzy adaptive differential evolution algorithm. Soft Comput.

[CR28] Lungarella M, Metta G, Pfeifer R, Sandini G (2003). Developmental robotics: a survey. Connect Sci.

[CR29] Nishimoto R, Namikawa J, Tani J (2008). Learning multiple goal-directed actions through self-organization of a dynamic neural network model: a humanoid robot experiment. Adapt Behav.

[CR30] Rocca P, Oliveri G, Massa A (2011). Differential evolution as applied to electromagnetics. IEEE Antennas Propag Mag.

[CR31] Rumelhart DE, McClelland JL, the PDP Research Group (1986) Parallel distributed processing: explorations in the microstructure of cognition, vol 1. MIT Press, Cambridge 10.1111/cogs.12148 25087578

[CR32] Rumelhart DE, Hinton GE, Williams RJ, Polk TA, Seifert CM (2002). Learning representations by back-propagating errors. Cognitive modeling.

[CR33] Sasaki K, Tjandra H, Noda K, Takahashi K, Ogata T (2015) Neural network based model for visual-motor integration learning of robot’s drawing behavior: association of a drawing motion from a drawn image. In: Proceedings of the IEEE international conference on intelligent robots and systems, pp 2736–2741

[CR34] Shaw P, Kabani NJ, Lerch JP, Eckstrand K, Lenroot R, Gogtay N, Greenstein D, Clasen L, Evans A, Rapoport JL (2008). Neurodevelopmental trajectories of the human cerebral cortex. J Neurosci.

[CR35] Shi Y, Eberhart R (1998) A modified particle swarm optimizer. In: Proceedings of the IEEE international conference on evolutionary computation, pp 69–73

[CR36] Solis FJ, Wets RJB (1981). Minimization by random search techniques. Math Oper Res.

[CR37] Storn R (1996) On the usage of differential evolution for function optimization. In: Proceedings of the biennial conference of the North American Fuzzy Information Processing Society, IEEE, pp 519–523

[CR38] Storn R, Price K (1995) Differential evolution—a simple and efficient adaptive scheme for global optimization over continuous spaces. Technical report, TR-95-012, ICSI, International Computer Science Institute, Berkeley, CA, USA

[CR39] Storn R, Price K (1997). Differential evolution–a simple and efficient heuristic for global optimization over continuous spaces. J Glob Optim.

[CR40] Takahashi K, Ogata T, Tjandra H, Yamaguchi Y, Sugano S (2015). Tool-body assimilation model based on body babbling and neurodynamical system. Math Probl Eng.

[CR41] Takahashi K, Ogata T, Yamada H, Tjandra H, Sugano S (2015b) Effective motion learning for a flexible-joint robot using motor babbling. In: Proceedings of the IEEE international conference on intelligent robots and systems, pp 2723–2728

[CR42] Tani J (2016). Exploring robotic minds: actions, symbols, and consciousness as self-organizing dynamic phenomena. Oxford series on cognitive models and architectures.

[CR43] Tani J, Nishimoto R, Paine R (2008). Achieving “organic compositionality” through self-organization: reviews on brain-inspired robotics experiments. Neural Netw.

[CR44] Törn A, Žilinskas A (1989). Global optimization. Springer series in lecture notes in computer science.

[CR45] Trelea IC (2003). The particle swarm optimization algorithm: convergence analysis and parameter selection. Inf Process Lett.

[CR46] Vesterstrom J, Thomsen R (2004) A comparative study of differential evolution, particle swarm optimization, and evolutionary algorithms on numerical benchmark problems. In: Proceedings of the IEEE Congress on evolutionary computation, vol 2, pp 1980–1987

[CR47] Wang Y, Wu X, Weng J, Lu BL, Zhang L, Kwok J (2011). Skull-closed autonomous development. Neural information processing. Lecture notes in computer science.

[CR48] Whittington JCR, Bogacz R (2019). Theories of error back-propagation in the brain. Trends Cogn Sci.

[CR49] Wieser E, Cheng G (2014) Predictive action selector for generating meaningful robot behaviour from minimum amount of samples. In: Proceedings of the IEEE international conference on development and learning and epigenetic robotics, pp 139–145

[CR50] Wieser E, Cheng G (2016) Progressive learning of sensory-motor maps through spatiotemporal predictors. In: Proceedings of the IEEE international conference on development and learning and epigenetic robotics, pp 43–48

[CR51] Wieser E, Cheng G (2018). A self-verifying cognitive architecture for robust bootstrapping of sensory-motor skills via multipurpose predictors. IEEE Trans Cogn Dev Syst.

[CR52] Yamashita Y, Tani J (2008). Emergence of functional hierarchy in a multiple timescale neural network model: a humanoid robot experiment. PLoS Comput Biol.

[CR53] Yamashita Y, Tani J (2012). Spontaneous prediction error generation in schizophrenia. PLoS Comput Biol.

[CR54] Yao X, Xu Y (2006). Recent advances in evolutionary computation. J Comput Sci Technol.

[CR55] Yao X, Liu Y, Lin G (1999). Evolutionary programming made faster. IEEE Trans Evol Comput.

